# Neutrophils are mediators of metastatic prostate cancer progression in bone

**DOI:** 10.1007/s00262-020-02527-6

**Published:** 2020-02-29

**Authors:** Diane L. Costanzo-Garvey, Tyler Keeley, Adam J. Case, Gabrielle F. Watson, Massar Alsamraae, Yangsheng Yu, Kaihong Su, Cortney E. Heim, Tammy Kielian, Colm Morrissey, Jeremy S. Frieling, Leah M. Cook

**Affiliations:** 1 Department of Pathology and Microbiology, University of Nebraska Medical Center, 985900 Nebraska Med Center, Omaha, NE 68192 USA; 2 Department of Cellular and Integrative Physiology, University of Nebraska Medical Center, Omaha, NE USA; 3 Department of Urology, University of Washington, Seattle, WA USA; 4 Tumor Biology Department, H. Lee Moffitt Cancer Center and Research Institute, Tampa, FL USA; 5 Department of Medical Education, California University of Science and Medicine, San Bernadino, CA USA

**Keywords:** Neutrophils, Bone, Metastasis, Prostate, Cancer, STAT5

## Abstract

Bone metastatic prostate cancer (BM-PCa) significantly reduces overall patient survival and is currently incurable. Current standard immunotherapy showed promising results for PCa patients with metastatic, but less advanced, disease (i.e., fewer than 20 bone lesions) suggesting that PCa growth in bone contributes to response to immunotherapy. We found that: (1) PCa stimulates recruitment of neutrophils, the most abundant immune cell in bone, and (2) that neutrophils heavily infiltrate regions of prostate tumor in bone of BM-PCa patients. Based on these findings, we examined the impact of direct neutrophil–prostate cancer interactions on prostate cancer growth. Bone marrow neutrophils directly induced apoptosis of PCa in vitro and in vivo, such that neutrophil depletion in bone metastasis models enhanced BM-PCa growth. Neutrophil-mediated PCa killing was found to be mediated by suppression of STAT5, a transcription factor shown to promote PCa progression. However, as the tumor progressed in bone over time, neutrophils from late-stage bone tumors failed to elicit cytotoxic effector responses to PCa. These findings are the first to demonstrate that bone-resident neutrophils inhibit PCa and that BM-PCa are able to progress via evasion of neutrophil-mediated killing. Enhancing neutrophil cytotoxicity in bone may present a novel therapeutic option for bone metastatic prostate cancer.

**Electronic supplementary material:**

The online version of this article (10.1007/s00262-020-02527-6) contains supplementary material, which is available to authorized users.

## Introduction

Approximately 90% of men with advanced prostate cancer (PCa)^1^ present with bone metastases. Bone is the preferential site for PCa metastasis and is associated with increased risk of fractures, spinal cord compression, and severe pain [1]. Although current standard-of-care bone- and osteoclast-targeted therapies, such as radium-223 and denosumab, delay tumor growth and cancer-induced bone disease, respectively, these have been unsuccessful in eliminating and preventing bone metastatic PCa (BM-PCa) growth in bone [2]. Patients with localized disease have a significantly better outcome than BM-PCa patients, with 5-year survival being reduced from nearly 100% for localized disease to less than 3% of bone metastatic disease [1]. To date, bone metastatic PCa (BM-PCa) remains incurable.

PCa cells thrive in the bone microenvironment by hijacking the coupled process of bone remodeling characterized by osteoclast-mediated bone degradation and osteoblast-mediated bone formation. Heightened bone turnover results in the release of bone sequestered factors such as transforming growth factor beta (TGFβ) [3] that promotes increased cancer cell survival and growth. While many studies have focused on the interplay between PCa cells, osteoblasts, and osteoclasts, it is increasingly evident that a large array of cell types in the bone microenvironment including mesenchymal stem cells (MSCs), hematopoietic stem cells (HSCs) and immune cells can contribute to the progression of these bone metastases [4].

Previously, we observed that PCa can regulate bone formation by recruiting MSCs, which in turn differentiate into bone-forming osteoblasts [5]. We also examined the differential effect of PCa cells on bone marrow MSC gene expression and observed a significant induction of interleukin (IL-8) in MSCs in response to PCa conditioned media. IL-8 is known to contribute to osteoclast formation but also is a potent chemoattractant for neutrophils [6]. Neutrophils and neutrophil precursors are the most abundant immune cell in bone, ~ 60% and ~ 40% of the human and mouse bone marrow compartment, respectively [7, 8]. Neutrophils are generated in the bone marrow at rates of 10^11^ cells daily and are regularly released into circulation [9]. Bodily infection or tissue damage results in a systemic gradient of chemokines being released, including IL-8 and CXCL5, which causes rapid neutrophil expansion and mobilization from the bone marrow or blood circulation into tissues followed by neutrophil secretion of a number of effector molecules, including bactericidal enzyme-containing granules, reactive oxygen species (ROS), and neutrophil extracellular traps (NETs), mesh-like scaffolds of decondensed DNA and granule enzymes [10]. In the past decade, emerging evidence has demonstrated a role for neutrophils in cancer progression, albeit conflicting, with data indicating both anti- and pro-tumor properties [11]. Recently, the existence of a pro-tumoral/“N2” and an anti-tumoral/“N1,” phenotype in the tumor microenvironment, has been described with emergence and dominance of the N2 subtype being regulated by TGFβ [12]. Surprisingly, given their abundance in the bone marrow, the role of neutrophils in the progression of bone metastatic prostate cancer has not been examined thus far.

In the current study, we established the presence of neutrophils in human BM-PCa biopsies and demonstrated that PCa cells stimulate neutrophil migration, oxidative burst, and NET formation, properties associated with neutrophil activation. Reciprocally, direct co-culture assays and live cell imaging revealed that neutrophils induce PCa apoptosis. In support of this, depletion of neutrophils in vivo accelerated the growth of BM-PCa in two independent mouse models of bone metastatic prostate cancer. This phenomenon appears to be regulated by neutrophil inhibition of STAT5 signaling in PCa. Additionally, we observed that the cytotoxic neutrophil phenotype was diminished as BM-PCa progresses in bone. Collectively, these findings are the first to demonstrate an anti-tumor role for neutrophils in the prostate tumor-bone microenvironment and demonstrate that BM-PCa progresses in bone via evasion of neutrophil-mediated PCa death.

## Materials and methods

### Tissues and cell lines

Luciferase-expressing cell lines were generated by the Cook Laboratory using a lentiviral luciferase reporter (Qiagen), according to protocol. To collect conditioned media (CM), cell lines were rinsed with Phosphate Buffered Saline (PBS) to remove serum, complete medium was replaced with serum-free medium and cells were incubated overnight. CM was collected 18–20 h later by brief centrifugation to remove cellular debris and stored at 4 °C until usage. To minimize media-based changes, all CM was collected in RPMI. Total protein content of CM was measured using BCA assay (ThermoFisher) to ensure equal protein concentrations for treating neutrophils. Fresh media was collected every 2 weeks for experimental use.

### Neutrophil isolation from bone marrow

Human bone marrow was processed using a modified Ficoll density centrifugation protocol. Briefly, bone marrow was washed in neutrophil isolation buffer at 7:1 ratio to bone marrow. This suspension was filtered with a 70-μM filter to remove bone fragments. Diluted bone marrow was slowly pipetted onto 15 mL of Ficoll-Paque in a 50-mL conical tube and centrifuged at 445 × g for 35 min at room temperature with no centrifuge brake. The granulocyte layer (the lower layer) was carefully removed and washed twice with buffer, and cells were counted for subsequent experiments. For mouse neutrophil isolation, mouse tibia and femurs were removed from male C57BL/6 mice. Bones were cleared of tissue and muscle, and the epiphysis was removed and discarded. A hole was made in the bottom of a 0.65 mL tube, and one bone was placed individually per tube. This tube was placed into a 1.5-mL tube for bone marrow collection, where it was then centrifuged at high speed for < 5 s. The bone marrow was re-suspended in 1 mL of neutrophil isolation buffer and filtered using a 70-μM filter. Bones from each mouse were pooled and counted, and the EasySep Mouse Neutrophil Enrichment (Stem Cell Technologies) protocol was followed, as per manufacturers’ instructions. Neutrophil purity was validated using flow cytometry for specific markers: human (CD11b^+^, CD14^−^, CD15^+^, CD16^+^, CD10^+^) and mouse (CD45^+^, CD11b^+^, Ly6G^hi^). All neutrophil described functional assays utilized mouse bone marrow neutrophils, and major findings were validated in human neutrophils.

### Cell culture media, reagents, and buffers

Neutrophil isolation buffer consists of 1X PBS, 2% FBS and 2 mM EDTA. RPMI complete media consists of RPMI (Hyclone), 10% FBS (Peak Serum), and 1% penicillin/streptomycin. DMEM complete media consists of DMEM (Hyclone), 10% FBS (Peak Serum), and 1% penicillin/streptomycin. Luciferase-expressing C42B and PAIII were supplemented with 10 μg of puromycin, and LNCaP with 5 μg puromycin (Gibco). For oxidative burst assays, cells were treated with either serum-free CM, lipopolysaccharide (LPS; 5 μg/ml, Sigma), or phorbol 12-myristate 13-acetate (PMA; 5 nM, Sigma) in serum-containing (10%) RPMI. For TβRI assays, primary neutrophils were incubated in CM supplemented with RepSox (5 nM; Selleckchem).

### Neutrophil migration assay

Permeable 24-well transwell migration chambers were used with a pore size of 5 μm (Costar; Ref # 3422). Primary neutrophils were isolated from mouse hind limbs and 1 × 10^5^ were seeded in 250 μL serum-free media in the inserts and 650 μL of conditioned media was added to the bottom of the respective wells, and then inserts were carefully lowered into the wells ensuring that no bubbles formed between the membrane and top of the conditioned media. Neutrophils were incubated for 1 h at 37 °C, then inserts were rinsed in 1x PBS and fixed overnight in methanol at − 20 °C. Fixed inserts were stained with hematoxylin for quantitation of number of migrating neutrophils per insert. Because ~ 90% of the neutrophils completely migrated through the inserts, neutrophils in the lower chamber media were counted using Trypan Blue Exclusion assay. For investigating the importance of IL8/CXCL1 in PCa-mediated neutrophil migration, bone-derived mouse neutrophils were pre-treated with an antibody to mouse CXCR2 (50 nM; Cayman Chemicals) for 30 min and CellTracker Green (Invitrogen) prior to addition to inserts and allowed to migrate for 1 h towards specific PCa CM. After inserts were removed, the wells were imaged using an EVOS FL Auto microscope (Invitrogen; AMAFD1000) at 10x to quantify neutrophils that completely penetrated the membrane.

### Immunofluorescence

Paraffin-embedded patient bone specimens and mouse tibia bone sections were dewaxed and hydrated through an alcohol gradient. Antigen retrieval for human and mouse specimens was performed using Tris–EDTA buffer (pH 9) in a pressure cooker for 6 min. Tissues were then blocked in 10% serum in 1X Tris-buffered saline (TBS) for 1 h prior to overnight incubation with primary antibodies (Cytokeratin (dilution 1:500), Sigma C2562; phospho-histone H3 (dilution 1:200), Cell Signaling 9701L; Neutrophil Elastase (dilution 1:200), Abcam ab68672; Myeloperoxidase (dilution 7.5 μg/ml), R&D AF3667). Following washes, species-specific secondary AlexaFluor antibodies were incubated 1:1000 on the tissues for 1 h at room temperature (A11029, A11036, A21206, and A11057). Fluorescent images were taken on a Zeiss Axio Imager.Z2 at 20x and quantified using ImageJ. Phospho-histone H3 was quantified as the number of positive cells compared to the total number of cells per image as determined by DAPI staining. STAT5 was quantified as the amount of positive STAT5 signal per total area of tumor tissue. A threshold for STAT5 signal was set for all images analyzed, and the percent of positive pixels was obtained for each image. Using cytokeratin, the total tumor area in pixels was also obtained. By multiplying the percentage of STAT5 and cytokeratin-positive pixels with the image area, we were able to obtain tumor area and STAT5 positive area. Dividing the calculated STAT5 area by the cytokeratin area yielded the percent of STAT5 per tumor area in each image.

### Flow cytometry

Isolated neutrophils were transferred to FACS buffer (2% FBS in 1X PBS) at 1 × 10^6^ cells in 200 μL. For tumor studies, bone marrow was flushed from tumor-bearing and saline-injected tibiae using a syringe and excess cells were further flushed out of the marrow with EasySep buffer. Cells were treated for 2–3 min in 1X Red Blood Cell Lysis Buffer (BioLegend) at room temperature, washed in 1X PBS, and counted for antibody staining. For staining, cells were incubated on ice with 1 μL per 10^6^ cells in mouse or human TruStain Fc block (Biolegend) for 10 min. Fluorophore-conjugated antibodies were added at a maximum of 1 μL per 10^6^ cells (Human-APC/Cy7-CD11b, PE/Cy7-CD14, FITC-CD15, PerCp-Cy5.5-CD10; Mouse- APC-CD45, FITC-CD11b, PE-Ly6G, PerCp-Cy5.5-Ly6C). Cell viability dye, Live/Dead (Invitrogen), was added at a concentration of 0.2 μL per 10^6^ cells. Stained cells were incubated with antibody on ice in the dark for 20 min and rinsed with 1X PBS. Cells were fixed by incubation in 1% formaldehyde in 1XPBS for 30 min in the dark and rinsed with 1X PBS. Prior to analysis, cells were reconstituted in FACS buffer. For all analyses, single cells were gated and marker expression analyzed in myeloid cells.

### Oxidative burst assay

Primary mouse neutrophils were isolated from whole bone marrow (EasySep). Neutrophils were incubated under sterile conditions in serum-free conditioned media from LNCaP, C42B, PC3, BPH, and RWPE-1 or appropriate serum-free base medium for an hour at 37 °C in flow cytometry tubes. For a positive control of neutrophil activation and oxidative burst, neutrophils were treated with lipopolysaccharide (LPS; 5 μg/mL) for 30 min in serum-containing RPMI. After incubation in prostate CM, dihydrorhodamine 1,2,3 (DHR123; 25 μM) was added to neutrophils in each CM condition, allowed to incubate for 30 additional minutes at 37 °C and cell fluorescence was analyzed via flow cytometry. Immediately prior to flow analysis, DAPI was added to each condition to measure cell viability. For a longitudinal analysis of oxidative burst, primary neutrophils were placed in a 96-well plate in prostate CM and DHR123 immediately added to each well. Green fluorescence/oxidized DHR was measured over time using an Incucyte S3 Live-Cell Analysis System Imager.

### Sytox green/NET formation assay

For analysis of NET formation, primary mouse neutrophils were incubated for 2 h in prostate CM (LNCaP, C42B, PC3, BPH, and RWPE-1) or complete RPMI supplemented with PMA (100 nM) as a positive control. Sytox Green dye (500 nM; Sigma) was added to each condition, and after 30 min, images were taken of each well by an EVOS FL Auto microscope. The number of green fluorescent secreted DNA traps/NETs was measured as a percentage of total cells to distinguish between dying cells that absorbed the Sytox Green dye. CM-treated neutrophils were compared to neutrophils incubated in serum-free media RPMI.

### Real-time qPCR

For analysis of neutrophils gene expression, RNA was isolated from neutrophils which had been incubated for 3 h at 37 °C, in PCa CM supplemented with 2% FBS. RNA was extracted using Trizol. RNA (1 μg) was used to synthesize cDNA using qSCRIPT Super mix (Quantabio) and PCR performed using Perfecta SYBR Green FastMix (Quantabio). PCR was run using Bio-Rad CFX Real-Time System. PCR conditions were as follows for all primer sequences (Supplemental Table 1): Step 1: 95° 30 s; Step 2: 95° 5 s, 57° 15 s, 72° 10 s, 95° 10 s (× 39 cycles); Step 3: Melt curve 65° 95° increments of 0.5° for 5 s.

### PCa co-cultures with neutrophils

Luciferase-expressing LNCaP or C42B cells were plated at 40,000 cells/well in a 24-well plate, in triplicate per condition. Twenty-four hours later, neutrophils were isolated, re-suspended in complete medium, and plated in direct contact with cancer cells at a ratio of 10:1 (neutrophils/cancer). After incubation overnight, neutrophils were removed, and cancer cell viability was measured with Trypan Blue Exclusion assay, using a hemacytometer.

### Incucyte S3 live-cell analysis and NanoLive

C42B and LNCaP co-cultures were performed using the above conditions, with modifications in cell density. C42B and LNCaP cells were plated at 9000 cells/well in a 96 well plate in triplicate, per condition. Mouse neutrophils were isolated the following day and plated at a ratio of 10:1 (neutrophils/cancer). The 96-well plate was placed into an Incucyte S3 Live-Cell Analysis System S3 Live-Cell Imager after neutrophils were added. Live-cell images were taken every 10 min and data quantified over a 24-h period. Three replicates of each cell line and condition were averaged, and data were analyzed using the Incucyte S3 Live-Cell Analysis System S3 analysis software (Essen biosciences). For imaging using NanoLive, C42B cells were plated in 6-well plates, and 24 h later, mouse bone marrow-derived neutrophils were added at a 10:1 neutrophil/cancer ratio and imaged for 1 h, as described.

### Real-time Glo MT cell viability assay

Neutrophils were incubated in triplicate at 100,000 per well in PCa CM with 2x Real-Time Glo reagent (Promega). MT Cell Viability Substrate and NanoLuc^®^ Enzyme were added in equal volumes to culture media to create the 2x Real-time Glo reagent. This media was added directly to the cells at time zero, and luminescence was read at the indicated time points using a luminometer.

### In vivo mouse models

Luciferase-expressing C42B, PAIII, and LNCaP cells were grown to confluence in complete media (base media, 10% fetal bovine serum, 1% penicillin/streptomycin). Cells were trypsinized and washed three times with 1X PBS and filtered using a 70-μM nylon filter. Cells were counted and reconstituted for injection of appropriate cell numbers per 20 μL volume per mouse (500,000 cells-LNCaP and C42B; 50,000-PAIII). Fox Chase SCID Beige male mice (Charles River) were anesthetized with isoflurane, and the right tibia was injected with 20 μL of cells. An equal volume of PBS was injected into the contralateral limb as a control for the intratibial injection. For imaging of tumor burden via bioluminescence, mice were given 10 μL/gram of D-luciferin (15 mg/mL) (Gold Bio) IP imaged using the IVIS Spectrum imager (Perkin-Elmer) on each day of antibody treatment. Luciferase signal was quantified 15 min after injection, using the Living Image Software per manufacturer’s instructions. Using bioluminescence intensity, mice were randomized into 2 groups to receive either (*n* = 5/group): a rat IgG2A isotype control antibody or Anti-Ly6G clone 1A8 (BioXcell) to ensure there were no differences in tumor burden at the start of neutrophil depletion. For LNCaP and C42B studies, mice received an intraperitoneal (IP) dose of 400 μg 1A8 on day 3 post-inoculation, and subsequent doses 200 μg twice per week to maintain low neutrophil numbers for the remainder of the study (for 2 weeks), based on previous experiments [13]. Depletion efficiency was determined by flow cytometry of bone marrow and spleen. For late-stage tumor depletion, C42B cells were intratibially injected in SCID Beige mice (*n* = 10/group) and after approximately 2 weeks were treated with isotype control or 1A8 antibody for the remainder of the study. For PAIII, mice received an initial dose of 100 μg antibody beginning on day 1 post-tumor inoculation and every other day for the remainder of the experiment. IVIS imaging was used for longitudinal measurement of tumor burden. To examine tumor-associated neutrophils throughout prostate cancer growth in bone, male SCID Beige mice (*n* = 15) were intratibially injected with 500,000 C42B cells in each limb and neutrophils were isolated from each tibia at 3 different time points after injection (week 1, week 2, and week 4). For control tumor-naïve neutrophil collections, additional mice (*n* = 5) were injected with saline. Mice were pooled per group and functional analyses performed: PCa co-culture assay, T cell suppression assay and cell viability.

### T cell proliferation assay

T cell proliferation assays were performed with neutrophils recovered from the bones of tumor-bearing and control mice as previously described [14]. Naïve CD4^+^ T cells were isolated from the spleens of C57BL/6 mice by negative selection (Biolegend) and labeled with eFluor670 cell proliferation dye (5 μM; ThermoFisher), according to the manufacturer’s instructions. T cells were plated at 1 × 10^4^ per well in a round bottom 96-well plate in RPMI-1640 supplemented with 10% FBS, 1% l-glutamine, 1% HEPES, 1% penicillin/streptomycin, 0.1% β-mercaptoethanol and 100 ng/ml recombinant mouse IL-2 (BioLegend). Control or tumor-associated neutrophils isolated at week 1 and 4 post-intratibial injection were added at 1:1 or 5:1 ratio (T cells:neutrophils) to CD4^+^ T cells subjected to polyclonal stimulation with anti-CD3/anti-CD28 Dynabeads (Gibco). TANs were isolated from tumor-bearing tibia, as described, and control neutrophils were isolated from saline-injected tibia.

### Trichrome staining

Tibias isolated from mice were fixed in 10% neutral buffered formalin then stored in 70% ethanol. Tibia were decalcified in 14% EDTA buffer for 2 weeks at 4 °C, changing the buffer every 2–3 days. Tibia were then embedded in paraffin and sectioned on a microtome to 5 μM sections. Tissues were cleared of paraffin in 2 changes of xylene and hydrated through an alcohol gradient. Slides were then incubated in Bouin’s fixative (Ricca, 1120-16) for 1 h at 55 °C. Slides were rinsed in running dH2O until clear, then stained with hematoxylin (Ricca, 3530-32) for 5 min. To blue the hematoxylin, slides were dipped in 1x TBS for 30 s, then stained in Gomori’s Trichrome solution (Volu-Sol, VXT-032) for 20 min. Slides were then transferred to freshly made 0.5% acetic acid solution for 2 min. The slides were rinsed in dH2O until clear, then dehydrated, cleared in xylene, and mounted with Permount (Fisher, SP15-500). Images were taken at 4x, 10x, and 20x using an EVOS FL Auto microscope (Life Technologies, AMAFD1000). Areas of osteogenesis were quantified as percent of trabecular bone over total marrow area using ImageJ starting 500 μm below the growth plate and continuing for 2 mm.

### Proteome profiler human phospho-kinase array

C42B and LNCaP cells were plated at 100,000 cells/well in a 6-well plate in triplicate, per condition. Mouse neutrophils were isolated the following day and plated at a ratio of 10:1 (neutrophils/cancer). 24 h later, the neutrophils were removed and protein was collected from lysed cancer cells for array analysis, performed per the manufacturer’s instructions (R&D Systems). Image J was used to measure array densitometry.

### Immunoblot analysis

Whole-cell extracts were lysed in RIPA buffer and Halt protease and phosphatase inhibitor cocktail (ThermoFisher) was added as per manufacturers’ instructions. Protein concentration was determined using BCA assay (ThermoFisher). Protein lysates were fractionated by SDS-PAGE and transferred to a nitrocellulose membrane using a transfer apparatus according to the manufacturer’s protocols (Bio-Rad). After incubation with 5% nonfat milk in 1X TBST for 1 h, the membrane was washed once with TBST and incubated with appropriate antibodies (STAT5 (1:1000; Cell Signaling #94205), β-actin (1:1000; Cell Signaling #4970) or GAPDH (1:2000; Cell Signaling #5174)) diluted in 1X TBST with 5% milk. Phosphorylated STAT5a/b (1:1000; Cell Signaling #4322) was diluted in 1X TBST with 5% BSA. Membranes were incubated with rotation at 4 °C overnight. After incubation, primary antibody was removed and membranes were washed three times for 10 min each in 1X TBST and incubated with a 1:5000 dilution of horseradish peroxidase-conjugated anti-rabbit antibodies (Cell Signaling) for 1 h at room temperature. Blots were washed with 1X TBST three times and developed with the Clarity Western System (Bio-Rad) according to the manufacturer’s protocols. Blots were imaged on a digital CCD developer (Azure Biosystems).

### Generation of STAT5 knockdown cells

STAT5A gene expression was reduced in C42B PCa cells using a STAT5A-targeted HuSH-29 shRNA lentiviral vector, which expresses red fluorescent protein (RFP). C42B cells were transfected using Lipofectamine, and RFP-positive cells were purified by FACS. As a control, C42B were transfected with a non-targeted scrambled sequence HuSH-29 shRNA vector. Three knockdown clones A, C and D were examined in direct co-cultures with mouse neutrophils in comparison to scrambled control C42B.

### Statistical analysis

Statistical analyses (*t* test, ANOVA) were performed using GraphPad Prism (GraphPad Software, Inc). Error bars represent standard error from the mean (SEM).

## Results

### Neutrophils co-localize with PCa cells in the tumor-bone microenvironment

**Fig. 1:**
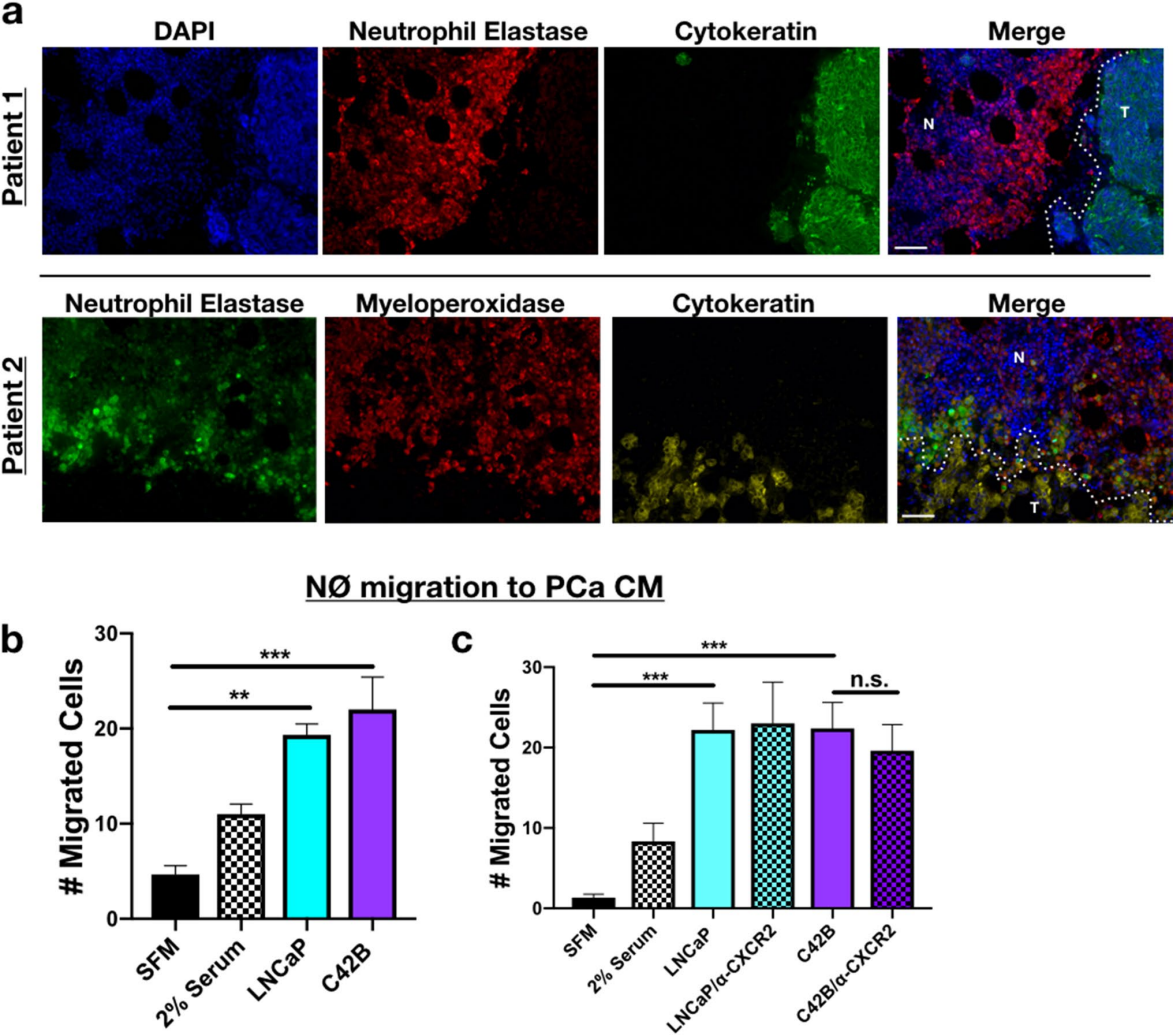
PCa recruitment of neutrophils. **a** Representative immunofluorescence (IF) of PCa and neutrophils in bone marrow of BM-PCa patients. Top: Patient 1—neutrophil elastase (NE; red) and epithelial marker, pan-cytokeratin (green), and nuclear marker, DAPI (blue); Bottom: Patient 2—neutrophil elastase (green), myeloperoxidase (red), cytokeratin (gold), DAPI (blue). “N” denotes normal bone marrow, “T” denotes a region of tumor in bone. Size bar = 50 μm. **b** Boyden chamber migration assay and shows number of neutrophils that migrated through the Boyden membrane into the lower chamber. Neutrophils were allowed to migrate toward specific conditions, for 1 h: serum-free media, serum containing 2% FBS, serum-free LNCaP conditioned media (CM), and serum-free C42B CM. **c** Neutrophil migration assay toward PCa media supplemented with an antibody to mouse CXCR2 (50 nM). Asterisks denote statistical significance (**p < 0.01, ***p < 0.001)

Previously, in examining the effect of bone metastatic PCa cells (C42B and PC3) on human MSC gene expression, we observed that IL-8 was highly induced in MSCs in response to prostate cancer derived factors [15]. IL-8 is a potent neutrophil chemoattractant, and we reasoned that the interplay between prostate cancer cells and bone marrow MSCs could result in the recruitment of neutrophils within the bone-PCa microenvironment. We examined whether this was the case in human samples of bone metastatic prostate cancer (*n* = 7). Immunofluorescence staining for the neutrophil-specific markers, neutrophil elastase (NE) and myeloperoxidase (MPO), revealed neutrophils proximal to prostate cancer cells (in Patient 1–5) (Fig. 1a, Supp. Figures 1A). This contrasted from areas of “normal” bone marrow tissue in which the neutrophils appeared to be more evenly distributed (Supp. Figure 1A). Similarly, in a mouse model of bone metastatic PCa (C42B), we noted NE-positive neutrophils at the tumor-bone interface (Supp. Figure 1B, bottom). These findings suggest that neutrophils in bone may be localized to regions of metastatic PCa. However, to determine whether PCa directly influences neutrophil recruitment, primary mouse bone marrow neutrophils were allowed to migrate toward either serum-free medium (SFM; as a negative control), SFM supplemented with 2% FBS (positive control), or media from human LNCaP (non/poorly metastatic PCa) cells or C42B (bone metastatic PCa cells derived from LNCaP) in modified Boyden chamber assays. We observed that both LNCaP and C42B similarly enhanced neutrophil recruitment (Fig. 1b) independently of CXCL1/8, the mouse homologues of IL-8, demonstrated by blockade of neutrophil CXCR2 (Fig. 1c). These findings collectively suggest that neutrophils in bone are recruited via PCa-derived soluble factors.

### PCa induces neutrophil oxidative burst and NET formation

**Fig. 2:**
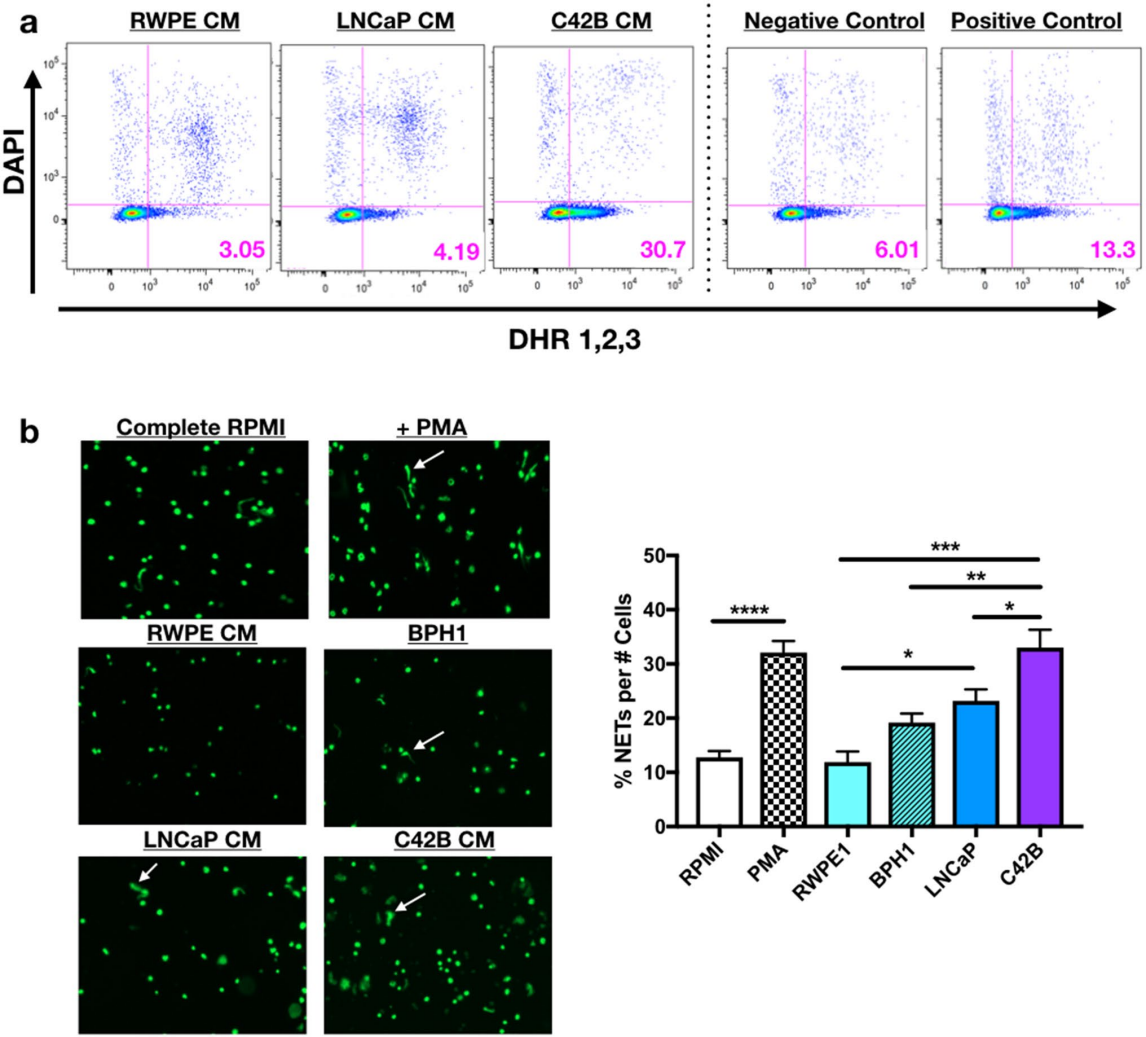
PCa induces neutrophil oxidative burst and NET release. **a** Neutrophils incubated for 3 h in conditioned media (CM) from RWPE, LNCaP and C42B cell lines supplemented with 5 nM dihydrorhodamine 123 (DHR123) and DAPI. Scatter plots show oxidative burst as expression of green fluorescence (x-axis) compared to DAPI (measure of viability) (y-axis) of CM-treated neutrophils compared to serum-free base media (negative control) and complete media, supplemented with 10% FBS and 5 μg/ml LPS (positive control). Images shown are representative from 1 of 4 repeat experiments. **b** Mouse bone marrow neutrophils incubated in CM for 2 h (*n* = 3 wells/media) and Sytox Green, DNA-binding dye for an additional 20 min. Images (left) were taken at a 10x objective under a GFP laser excitation using the EVOS Auto Light Microscope. White arrows denote “string-like” DNA-containing NETs. Quantitation of NETs (graph) were defined as the percentage of NETs per total cell number per well. Asterisks denote statistical significance (**p < 0.01, ***p < 0.001, *p *< 0.0001)

Based on our evidence of PCa-mediated neutrophil recruitment, we next examined the impact of PCa on neutrophil function. Oxidative burst is a classical neutrophil cytotoxic response against pathogens. However, in the tumor microenvironment, oxidative burst has been shown to both inhibit tumor growth and to promote tumor growth indirectly via suppression of T cell activation [16]. To determine the impact of BM-PCa on neutrophil oxidative burst, primary bone marrow-derived mouse neutrophils were treated with PCa-derived CM from (1) poorly metastatic LNCaP or (2) bone metastatic C42B, in comparison with non-malignant RWPE prostate epithelial cells. For negative and positive controls, neutrophils were treated with either SFM or serum-containing medium supplemented with LPS (or PMA), an inducer of oxidative burst. Reactive oxygen species (ROS), as a measure of oxidative burst, was measured by dihydrorhodamine (DHR) 1,2,3, a dye that passively diffuses into cells and, upon oxidation, emits green fluorescence as a readout for ROS production [17]. Surprisingly, we observed that neutrophils exposed to bone metastatic C42B CM for 2 h produced more ROS than those treated with LNCaP (~ sevenfold more) and RWPE cell line media (~ sixfold more) and 2.5-fold more than the positive control, LPS (Fig. 2a, Supp. Figure 2A). However, longitudinal measurement of oxidative burst revealed that all of the prostate cancer cells and benign prostate hyperplasia (BPH-1) cells induced neutrophil oxidative burst, although at different time points. Specifically, LNCaP quickly activated neutrophils within the first 30 min of treatment, whereas PC3- and C42B-treated neutrophils produced the most ROS after 2 h of treatment (Supp. Figure 2B).

As an additional measure of PCa-mediated neutrophil activation, neutrophil extracellular trap (NET)-formation was examined. NETs are sticky mesh-like secretions of DNA combined with granule enzymes released by activated neutrophils to sequester and degrade bacterial pathogens and are stimulated by enzymes, myeloperoxidase and neutrophil elastase, which are also involved in neutrophil oxidative burst [18]. To examine the impact of PCa on NET secretion, primary bone marrow mouse neutrophils were incubated in prostate CM for 2 h and Sytox Green was added to the media to bind extruded DNA. Similar to the oxidative burst assay, PCa media significantly induced neutrophil secretion of NETs; interestingly, LNCaP and C42B media induced significantly more NETs than non-malignant prostate cells, RWPE-1 (two and threefold, respectively; *p *< 0.05, 0.01) and BPH-1 (Fig. 2b). These findings demonstrate that PCa factors induce activation and, specifically, ROS and NET production of bone marrow neutrophils.

### Neutrophils regulate PCa growth in vitro independently of TGFβ

**Fig. 3:**
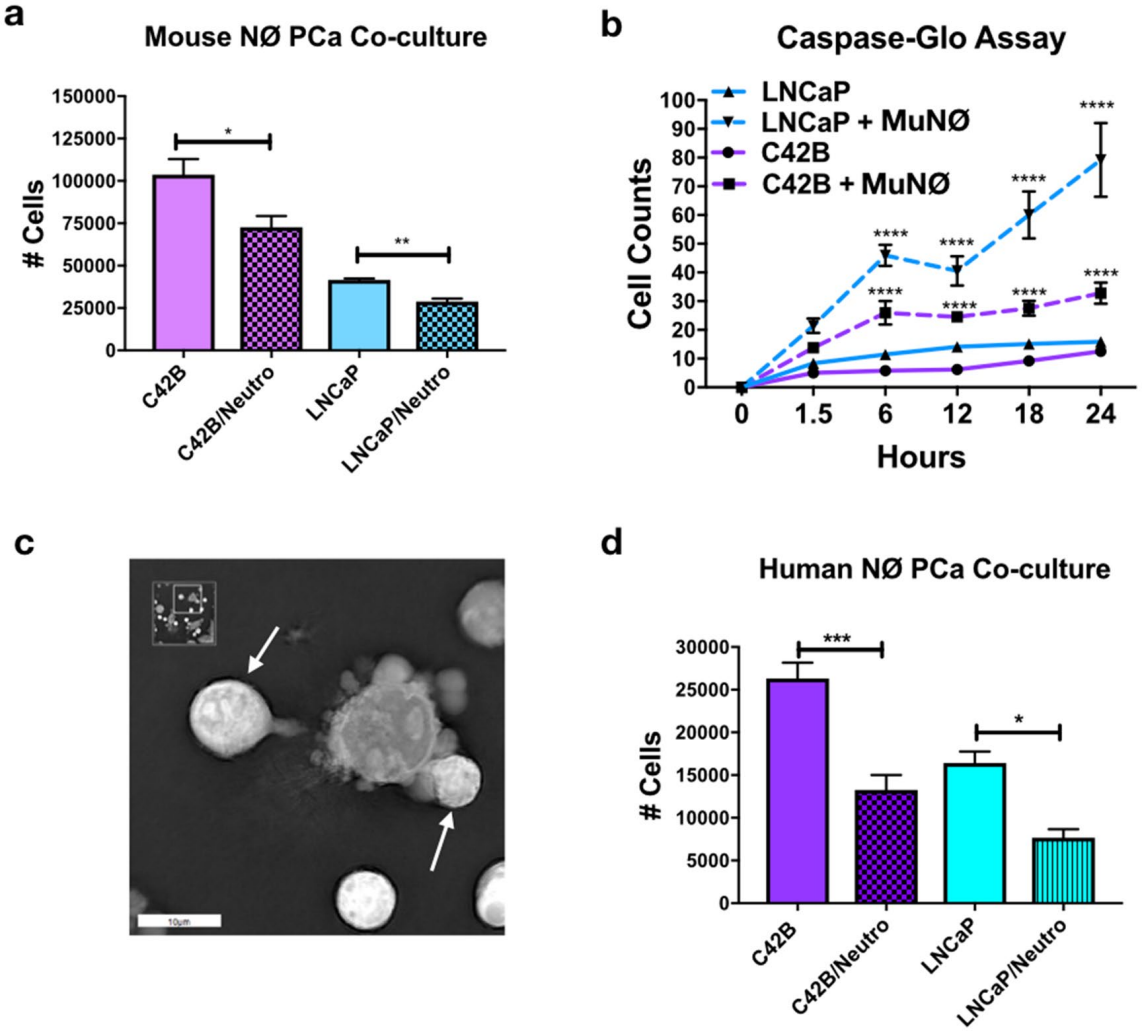
Neutrophils regulate PCa growth in vitro. **a** LNCaP or C42B cell numbers were quantified by Trypan Blue Exclusion assay 24 h after direct co-culture with mouse bone marrow-derived neutrophils plated at a 10:1 ratio of neutrophil to cancer (*n* = 3). Asterisks denote statistical significance (*p < *0.05*, **p < *0.01*). **b** Caspase activity quantification during Incucyte S3 Live-Cell Analysis System imaging. Incucyte S3 Live-Cell Analysis System caspase-3/7 green reagent was added to neutrophil–PCa co-cultures when neutrophils were added to cancer cells. Quantification of cells was calculated based on presence of the green Caspase dye. Solid lines are PCa in culture; dashed lines are PCa cells cultured with neutrophils. Asterisks denote statistical significance (****p < *0.0001*) (*n* = 3) for each condition. **c** Snapshot image of mouse neutrophils with cultured C42B cells imaged using NanoLive 3D Explorer for 1 h. White arrows denote neutrophils surrounding a C42B cell with membrane blebbing. Asterisks denote statistical significance (*p < *0.05*, ***p < *0.001*) **d** Trypan Blue quantitation of LNCaP and C42B 24 h after culture with human bone marrow neutrophils

Previous evidence in other cancer types has demonstrated that cancer-induced neutrophil ROS and NETs contribute to tumor proliferation, demonstrated in other cancer types [19–21]. However, the consequences of neutrophil activation and direct neutrophil–BM-PCa interactions on tumor growth have not been fully explored. To test this, neutrophils were isolated from bone marrow of C57BL/6 mice and cultured in direct contact at a 10:1 neutrophil/PCa ratio with LNCaP and C42B cells in vitro for 24 h. This ratio was based on the relative numbers of neutrophil/PCa in preclinical bone metastasis models from our group and was determined to induce the most significant impact on PCa growth (data not shown). Cell counts showed that mouse neutrophils significantly reduced C42B and LNCaP growth compared to PCa cultured with no neutrophils (Fig. 3a). To determine whether the impact on growth was due to heightened apoptosis, LNCaP and C42B were pre-incubated with Caspase 3/7 Green Apoptosis Assay Reagent and then cultured in contact with neutrophils for 24 h. As early as 6 h after neutrophils were added, green fluorescence (indicative of increased caspase activity) of LNCaP and C42B was increased ~ fourfold (LNCaP: 46 with neutrophils, 11.4 LNCaP alone; C42B: 26 with neutrophils, 5.8 C42B alone). At 24 h, there was a significant increase in apoptosis in both LNCaP (at 24 h, ~ fivefold more than LNCaP alone, *p *< 0.0001) and C42B (at 24 h, ~ threefold more than C42B alone; *p *< 0.0001) (Fig. 3b). There were surprisingly more apoptotic LNCaP than C42B cells despite similar reductions in both LNCaP and C42B numbers observed in Trypan Blue assays and in Incucyte S3 Live-Cell Analysis System-measured cell confluency (Supp. Figure 3). Real-time holotomography microscopy (Nanolive) further supports the PCa apoptotic inducing roles of neutrophils (Fig. 3c and (Supp. Movie 1). Previous findings have demonstrated differences in mouse and human neutrophil cytotoxicity, and thus we examined the impact of human neutrophils (isolated from human bone marrow aspirates) on LNCaP and C42B growth and found that human neutrophils significantly reduced both LNCaP and C42B growth by ~ 2.5-fold (*p *< 0.001; *p *< 0.05) (Fig. 3d) demonstrating that bone marrow neutrophils are cytotoxic to BM-PCa.

Based on evidence demonstrating that TGFβ promotes the emergence of pro-tumoral neutrophils [12], we examined the impact of PCa on neutrophil TGFβ ligand and receptor gene expression. Real-time qPCR revealed that the bone metastatic C42B-derived soluble factors significantly increased mouse neutrophil expression of TGFβI, TβRI, and TβRII by ~ twofold (Supp. Figure 4A) and human neutrophil TβRI/ALK5 expression ~ fivefold (*p *< 0.05) (Supp. Figure 4D) in support of previous findings [12]. Based on evidence showing that cancer CM can increase neutrophil survival [11], we examined the impact of BM-PCa and TβRI expression on neutrophil viability. LNCaP media had the largest impact on neutrophil survival where 50% of mouse neutrophils in LNCaP media were still viable at 18 h compared to SFM (i.e., baseline) and C42B (~ 25% viable cells for both conditions) (Supp. Figure 4B). Likewise, human neutrophils exhibited similar responses in viability; however, luminescence of human neutrophils in PCa media increased between 24 and 48 h suggesting that there were more cells at those time points (Supp. Figure 4E). Inhibition of neutrophil ALK5 activity using a small molecule kinase inhibitor, RepSox, further increased neutrophil numbers and viability. ALK5 inhibition had a moderate impact on mouse neutrophil-mediated PCa apoptosis but no effect on human neutrophils (Supp. Figure 4C, F). These findings reveal that PCa-induced TβRI is not a major mediator of neutrophil-mediated PCa death but that both mouse and human neutrophils respond similarly to PCa.

### Neutrophil depletion enhances growth of bone metastatic PCa in vivo

**Fig. 4:**
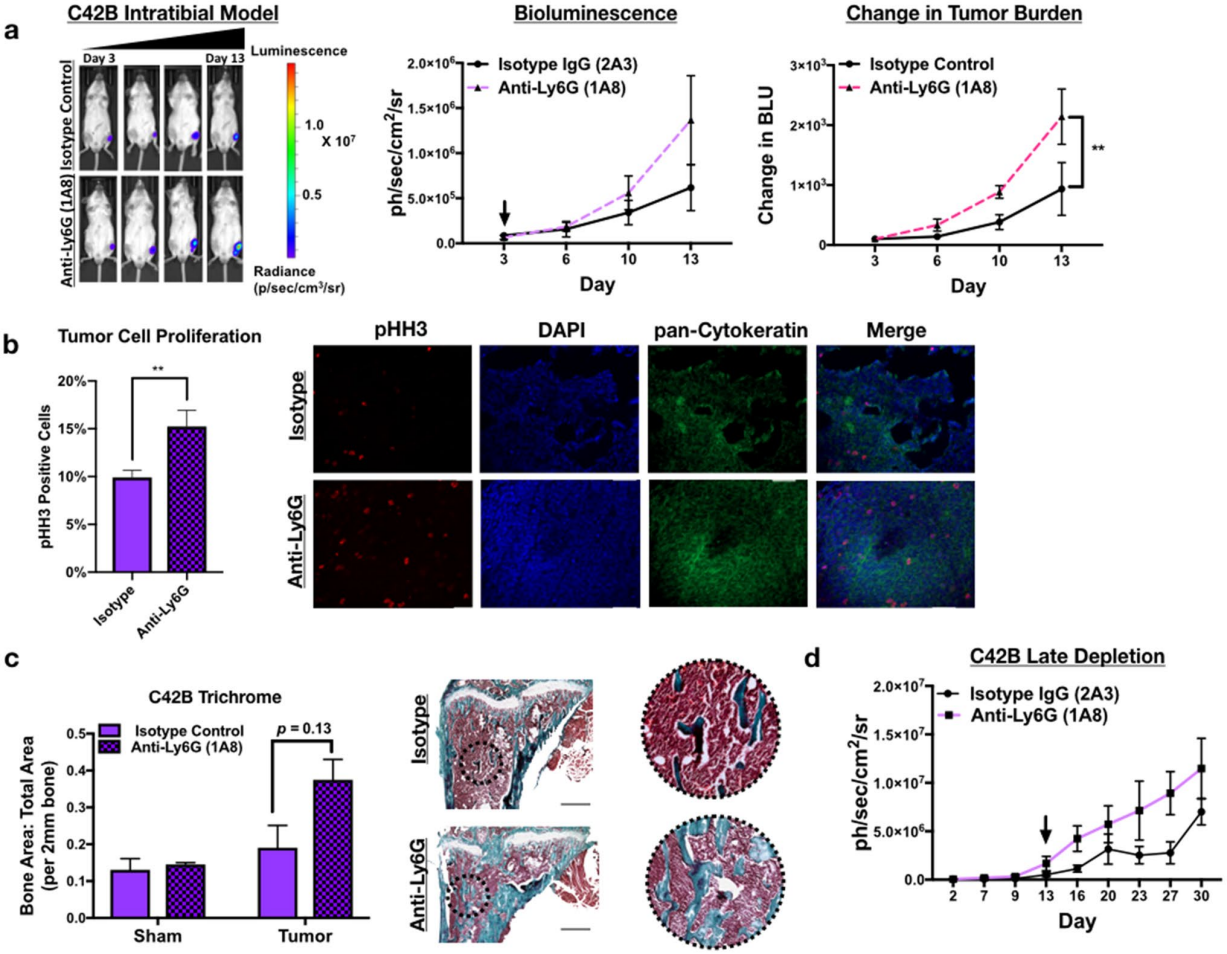
Neutrophils mediate growth of bone metastatic PCa in vivo. **a** Luciferase-expressing C42B cells were injected intratibially into SCID Beige mice. Representative images, quantitation of bioluminescence (left graph), and change in tumor bioluminescence compared to start of study (right graph) of C42B (*n* = 5/group) treated with isotype control or anti-Ly6G 1A8 antibody 3 days after inoculation. Arrow denotes start of neutrophil depletion. **b** Quantitation (graph on left) and representative IF images (right) of C42B (denoted by cytokeratin (green)) expression of phosphorylated Histone H3 (red) in bone. Nuclei are stained with DAPI (blue). Size bar = 20 μm. Asterisks denote statistical significance (**p < *0.01*) **c** Trichrome quantitation of saline-injected (sham) and tumor-inoculated tibia of isotype vs. anti-Ly6G-treated mice in representative graph (left); representative images (left) show Trichrome stain of tumor tibia with magnified inset. Type I collagen/bone is stained blue. Size bar = 500 μm. **d** Bioluminescence of luciferase-expressing C42B cells. C42B-luc cells were injected intratibially into SCID Beige mice (*n* = 5/group) and neutrophil depletion (via anti-Ly6G (1A8)) or isotype control treatments were started 2 weeks post-tumor inoculation (at arrow), denoted as late depletion

To determine the effect of neutrophils in the prostate tumor-bone microenvironment, luciferase-expressing C42B were injected intratibially into male SCID Beige mice. Three days after inoculation, mice were randomized based on bioluminescence signals and randomized into control (2A3 isotype control) or neutrophil-depleted (anti-Ly6G antibody; clone 1A8) groups (*n* = 5/group). The 1A8 antibody selectively depletes Ly6 G^+^ cells, without depleting other Ly6G-negative myeloid cell populations (such as macrophages) [22]. Neutrophil depletion in mouse tibia and spleen was confirmed by flow cytometry (Supp. Figure 5). Using bioluminescence as a readout for tumor growth, we observed that 1A8 increased C42B growth rates over time compared to controls (Fig. 4a) (2.3-fold, *p *< 0.001). In support of this result, immunohistochemical analysis of 1A8-treated mice showed a significant increase in the proliferation marker phosphorylated histone H3 (pHH3) in cytokeratin-positive C42B compared to the control group (Fig. 4b). This increase in tumor volume also enhanced cancer-induced bone formation as there was no difference in the bone volume of saline-injected/“sham” limbs (Fig. 4c).

Previous studies showed that pro-tumoral neutrophils emerge in the tumor microenvironment as tumor burden increases [12]. To examine this possibility in BM-PCa, C42B cells were injected intratibially in SCID Beige mice and after 2 weeks, neutrophils were depleted using 1A8. Similar to early stage neutrophil depletion, 1A8 increased tumor burden compared to isotype control-treated mice (Fig. 4d). Specifically, tumor burden at Day 27 was ~ threefold higher in neutrophil-depleted mice compared to isotype control (bioluminescence: 8.9 × 10^6^ (1A8) vs. 2.8 × 10^6^(control)) suggesting that neutrophils in the tumor-bone microenvironment are predominantly anti-tumoral and that depletion of these populations accommodates BM-PCa growth in bone.

**Fig. 5:**
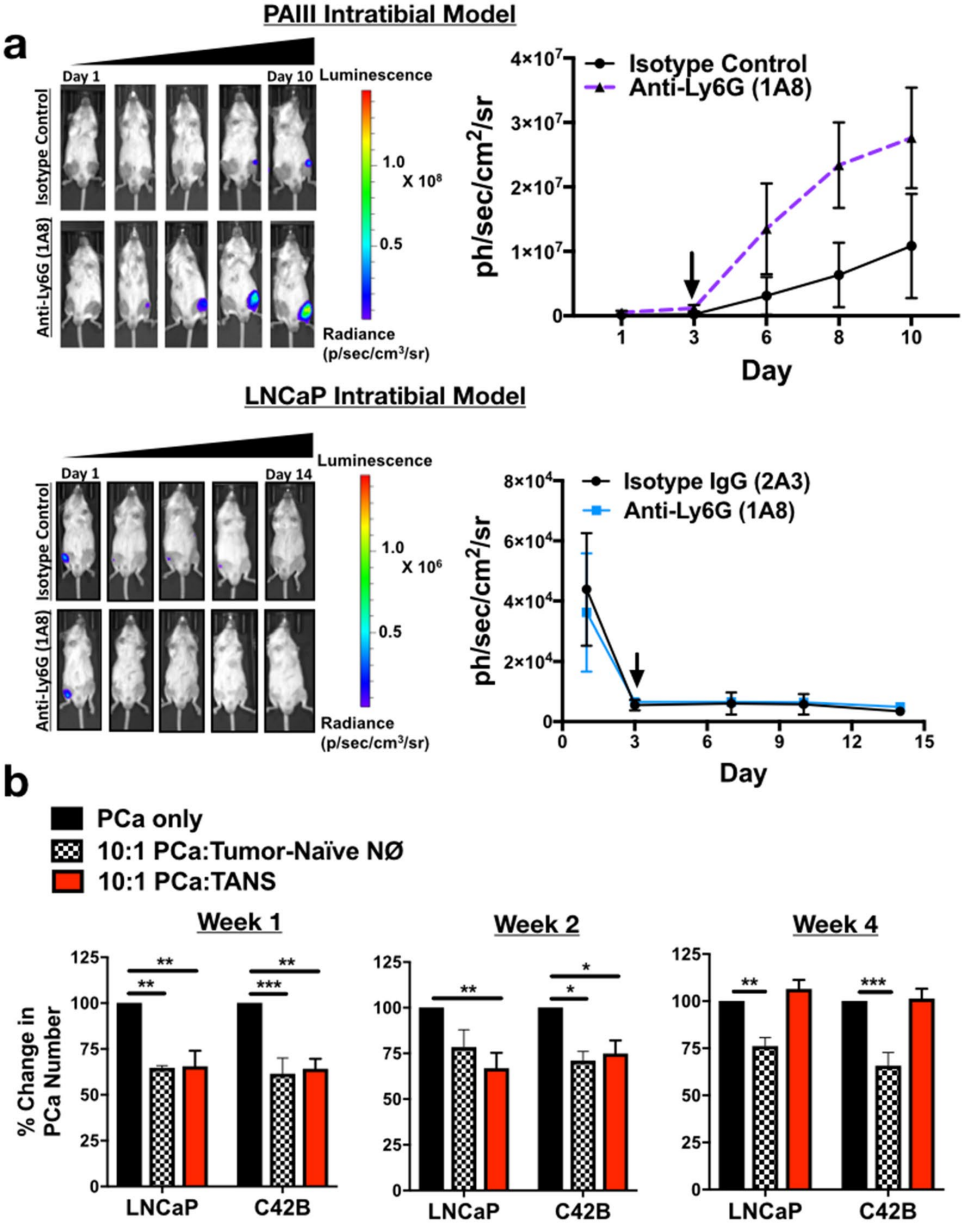
Neutrophil depletion is permissive for bone metastatic but not non-metastatic prostate cancer growth in bone but lose cytotoxicity with tumor progression. **a** Top, PAIII cells were injected intratibially into SCID Beige mice (*n* = 5/group) and treated with anti-Ly6G (1A8) or isotype control antibody. Bioluminescence is shown in representative images (left). Graph represents change in bioluminescence/growth rate from day of neutrophil depletion start at Day 3 (right). Arrow in graph denotes start of neutrophil depletion. Bottom, bioluminescence of luciferase-expressing LNCaP cells. LNCaP-luc were injected intratibially into SCID Beige mice (*n* = 5/group) and neutrophil depletion started 3 days post-tumor inoculation. Arrow indicates start of treatment. **b** C42B cells (or saline as a control) were injected intratibially into male SCID Beige mice and mice euthanized at Weeks 1, 2 and 4 post-injection for isolation of bone neutrophils. Control neutrophils (from saline-injected tibia; denoted as NØs) and tumor-associated neutrophils (TANs) were cultured ex vivo with LNCaP and C42B cell lines for 24 h and counted via Trypan Blue Exclusion assay. Graphs show percent change in cell number after 24 h of culture with neutrophils. Asterisks denote statistical significance (*p < 0.05, **p < 0.01, ***p < 0.001)

For comparison in another bone metastatic PCa model (i.e., prostate tumor-derived cells that grow in bone), we examined the importance of neutrophils in the growth of PAIII rat adenocarcinoma, which have been shown to induce osteolytic and osteogenic bone lesions in vivo. Similar to our findings with the C42B model, depletion of neutrophils in the PAIII intratibial model increased PAIII growth in bone which showed a 3.5-fold increase in luminescence of neutrophil-depleted mice at Day 8 (Fig. 5a, top). We demonstrated that these findings were not simply due to the PCa “filling in” the additional bone marrow space provided by the neutrophil depletion as previously shown [23], by depleting neutrophils from the intratibial model of LNCaP, cells considered poorly metastatic since they do not typically grow in bone [24]. Despite the ability of neutrophils to kill LNCaP in vitro, LNCaP did not grow in bone even in the absence of neutrophils (Fig. 5a, bottom) in the bone marrow space.

Although these findings suggest that neutrophils in the tumor-bone environment are predominantly cytotoxic, we next examined the impact of BM-PCa on neutrophil function throughout tumor progression in bone. To do this, C42B cells were injected intratibially in SCID Beige mice and bone marrow neutrophils/tumor-associated neutrophils (TANs) were isolated from bone at Weeks 1, 2, and 4 post- C42B injection for the following functional analyses: impact on PCa growth in PCa-neutrophil co-culture assays, neutrophil viability, NET formation and T cell suppression. TAN function was compared to neutrophils isolated from mice injected intratibially with saline. As previously seen, both control neutrophils and TANs inhibited LNCaP and C42B growth at Weeks 1 and 2 (~ 2.5-fold, ~ 30% reduction in growth); however, TANs isolated from marrow 4 weeks post-injection did not induce PCa cell death and appeared to have no impact on PCa growth (Fig. 5b). Further, Week 4 TANs showed different functional characteristics compared to Week 1 and 2 TANs and exhibited prolonged cell viability (40% viable cells at 24 h and 20% viable at 48 h; *p *< 0.001) compared to complete cell death of control neutrophils and Week 1 and 2 TANs by 24 h (Supp. Figure 6A). Additionally, Week 4 TANs produced significantly less NETs implicating reduced activation (Supp. Figure 6B).

Finally, we examined the T cell suppressive properties of control bone marrow neutrophils compared to TANs at Weeks 1 and 4 post-tumor injection. Naïve CD4^+^ T cells were isolated from spleen of C57BL/6 mice and cultured directly with control neutrophils or TANs along with CD3 and CD28 Dynabeads to stimulate T cell proliferation. Surprisingly, both tumor-naïve control neutrophils and TANs were significantly suppressive of T cell proliferation such that there were fewer proliferating/more non-proliferating T cells compared to the positive control T cells with no neutrophils (~ two- to threefold difference; *p *< 0.01). However, by Week 4 post-injection, TANs were ~ twofold less suppressive of T cells than the control neutrophils (Supp. Figure 6C). These findings revealed that: (1) bone marrow neutrophils cytotoxic to PCa are also suppressive of T cell proliferation and (2) that TANs in the tumor-bone microenvironment are functionally altered throughout tumor progression. Collectively, these results demonstrate that bone marrow neutrophils suppress PCa growth in vivo and in vitro and, specifically, metastatic PCa is able to grow in bone, in part, by evading neutrophil-mediated cell killing.

### Bone-resident neutrophils induce PCa apoptosis via inhibition of Stat signaling

**Fig. 6:**
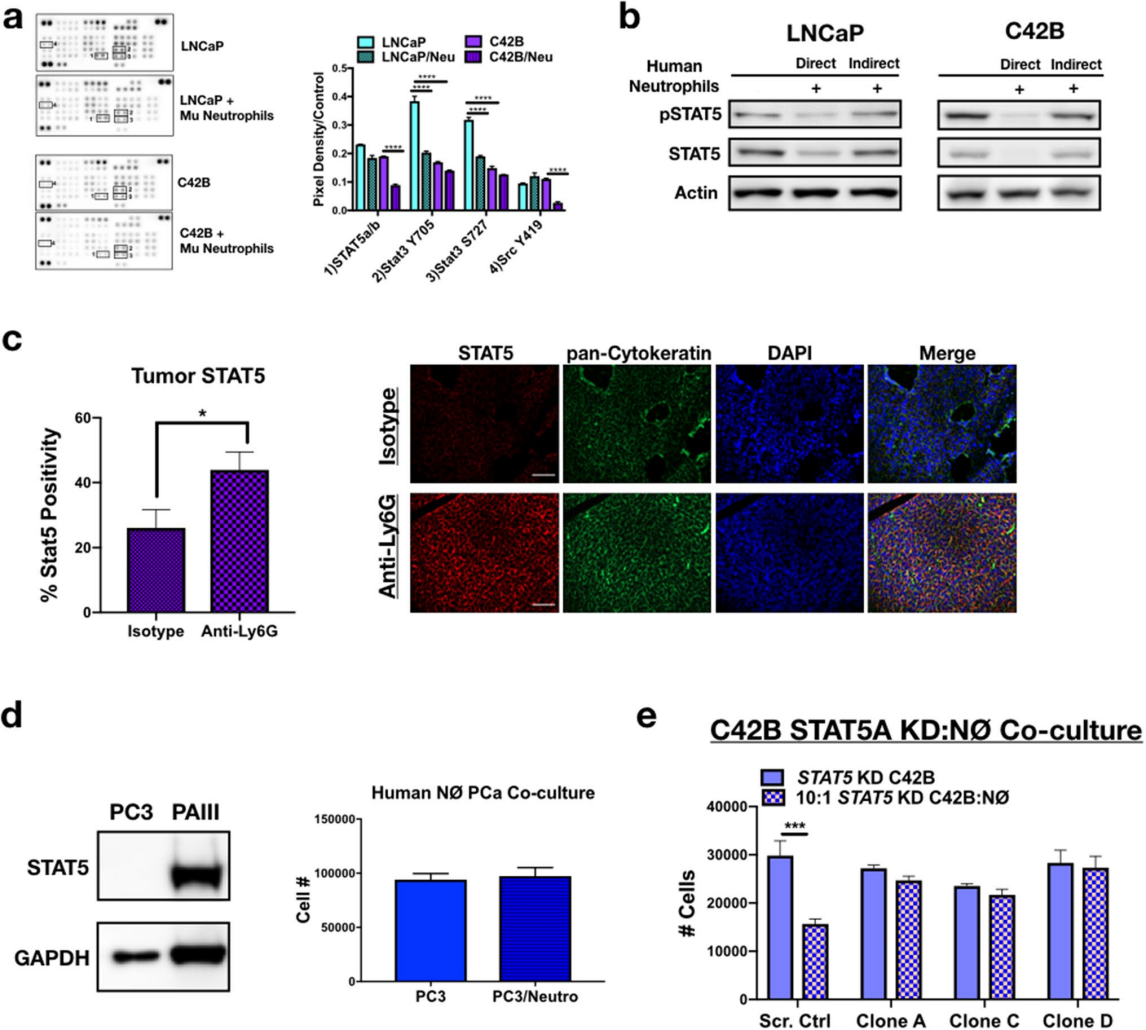
Neutrophils induce PCa death via inhibition of STAT5. **a** Phospho-kinase protein array of LNCaP and C42B cells cultured alone or with mouse bone marrow-derived neutrophils (denoted as Mu neutrophils). Boxes correspond to the genes labeled in. Three samples of each condition were pooled, and 100 μg of protein loaded onto the array. Proteins are displayed in duplicate. Average dot pixel density was averaged per protein target, background density subtracted, and normalized to the reference control per blot. Densitometry analysis was performed using Image J software. Graph represents pixel density quantitation of pSTAT5, pSTAT3, and pSrc. Asterisks denote statistical significance (****p < 0.0001). **b** STAT5 western blot of whole-cell PCa (30 μg protein) lysates from direct and indirect co-cultures of human neutrophils with LNCaP (left) and C42B (right) cells. For indirect co-cultures, modified Boyden chamber assay was utilized. **c** Immunofluorescence quantitation is the percentage of STAT5-positive C42B per total cell number (left) and representative images (right) of Total STAT5 (red) in C42B bone metastases (*n* = 5). C42B tumor cells in bone are denoted by cytokeratin (green), and nuclei are stained by DAPI (blue). Size bar = 20 μm; asterisk denotes statistical significance (*p < 0.05). **d** Left, Western blot analysis of Stat5 in PC3M compared to PAIII. Right, PC3M cell counts 24 h after direct co-culture with human bone marrow neutrophils. **e** Direct co-culture of bone-derived mouse neutrophils and C42B cells expressing: a non-targeted scrambled sequence shRNA (Scr. Ctrl) or STAT5A shRNA plasmids (denoted STAT5 knockdown (KD) clones A, C, and D). Graph shows the total number of C42B cells 24 h after co-culture measured by Trypan Blue Exclusion assay. Asterisk denotes statistical significance (***p < 0.001)

We next considered the molecular pathways being targeted by neutrophils that could promote PCa cell death. Unlike the effects noted with TβRI inhibition on neutrophil viability, pre-incubating neutrophils with the TβRI inhibitor RepSox and subsequent direct co-culture with PCa revealed no impact on PCa viability (Supp. Figure 4B and 4E). We therefore employed a more global approach. Using a receptor tyrosine kinase phosphorylation array, we examined the status of LNCaP and C42B kinase signaling in response to neutrophils and observed a significant reduction in the phosphorylation of several kinases important for prostate cancer cell survival (Fig. 6a and Supp. Figure 7A). Specifically, to distinguish between mouse and human proteins, neutrophils isolated from mouse bone marrow were cultured with the human PCa cells, protein was isolated from the PCa cells, and specific protein changes were validated in human neutrophil co-cultures, using human neutrophils isolated from bone marrow in culture with the human PCa cell lines. Phosphorylation of the non-receptor kinase Src was significantly reduced 5.5-fold in C42B cultured with neutrophils (0.11 vs. 0.02; *p *< 0.0001), compared to neutrophil-treated LNCaP (no change in Src). Additionally, phosphorylation of Src downstream target, STAT5, was significantly inhibited in C42B (0.19 vs. 0.09; *p *<0.0001). Interestingly, mouse neutrophils significantly inhibited Stat3 phosphorylation in LNCaP, whereas Stat3 was unaffected in C42B suggesting that Stat pathways may be critical mediators of BM-PCa growth in bone (Fig. 6b). To determine whether STAT5 phosphorylation is related to neutrophil direct contact with PCa (and to confirm that the STAT changes were not species specific), we isolated neutrophils from human bone marrow and performed direct and indirect (using modified Boyden Chamber) co-culture assays. Although neutrophils reduced PCa numbers in indirect co-culture, total and phosphorylated STAT5 was inhibited more with direct compared to indirect co-culture conditions (Supp. Figure 7B). In support of these findings, IF of neutrophil-depleted C42B intratibial tumors revealed significantly more STAT5 expression compared to isotype control-treated mice (Fig. 6c). Interestingly, human neutrophils had no impact on the growth of PC3 PCa, which are STAT5 negative compared to STAT5-expressing PAIII cells (Fig. 6d).

Previously STAT5 has been shown to promote metastatic prostate cancer growth, such that STAT5 inhibition results in reduced growth of BM-PCa cells [25]. To determine whether STAT5 is important for neutrophil-induced PCa death, we reduced C42B expression of STAT5 via shRNA. Based on qPCR analysis of C42B gene expression after neutrophil co-culture which showed a significant reduction in STAT5A compared to STAT5B (Supp. Figure 8A), we focused on STAT5A inhibition. However, knockdown of STAT5A gene expression via a STAT5A shRNA also reduced gene expression of STAT5B (Supp Figs. 8B, C), which is ~ 92% homologous to STAT5A. Mouse bone marrow neutrophils had no impact on C42B with reduced STAT5 expression (STAT5 KD C42B) compared to control C42B e.g., expressing a non-targeted scrambled sequence shRNA (Fig. 6e). Despite overlap in STAT5A/B targeting, there was little impact on STAT3, another STAT targeted by mouse neutrophils in LNCaP (Fig. 6a and Supp. Figure 8D), demonstrating the specificity of the gene knockdown to STAT5. These findings revealed that neutrophil-mediated PCa apoptosis is dependent on PCa STAT5 expression. Further analysis will be required to define the specific mechanisms involved in neutrophil-mediated STAT5 inhibition.

### Discussion

The majority of PCa-associated deaths are due to the development of advanced stage castration-resistant disease, which typically progresses to incurable bone metastatic PCa (BM-PCa). Current standard immunotherapies targeting T cell activation (e.g., checkpoint inhibition blockade) have been beneficial for some advanced stage cancers but have been less reliable for patients with bone metastatic PCa ranging from complete response to no response at all [26, 27]. However, given the abundance of immune cells in the BM-PCa bone microenvironment, there is a significant need for defining the role of immune populations in regards to disease progression. With the abundance of neutrophils in the bone microenvironment and existing evidence of TGFβ driving pro-tumoral neutrophil function, we hypothesized that tumor-associated neutrophils in bone contribute to PCa progression. This hypothesis was supported by evidence of neutrophil recruitment toward PCa media and patient bone biopsies showing regions of neutrophil localization to the tumor interface and PCa recruitment of bone marrow-derived neutrophils in vitro (Fig. 1). Surprisingly, our studies demonstrated that PCa activates bone-derived neutrophils, which are cytotoxic to PCa and directly induce BM-PCa apoptosis via targeting of STAT5. These are the first studies, to date, to show the impact of direct neutrophil–PCa interactions on metastatic growth in bone.

Our findings showed that neutrophils directly inhibited STAT5 expression in C42B, which appears to be necessary for neutrophil-mediated PCa apoptosis. Recent studies showed that STAT5 is a mediator of growth and invasion of bone metastatic PCa where siRNA-mediated STAT5 inhibition significantly increased PCa Caspase 7 expression and apoptosis [25, 28]. Additional studies showed that STAT5 expression is increased in aggressive prostate cancer and inhibition of STAT5 was able to sensitize BM-PCa xenografts to radiation through blockade of STAT5-mediated DNA repair [29, 30]. Our studies revealed that neutrophil-mediated PCa death is due to specific inhibition of STAT5 expression supporting prior evidence that STAT5 is a potential target for preventing progression of aggressive, metastatic PCa. However, STAT5 is a critical factor for neutrophil function and T cell differentiation [31, 32]; thus, STAT5-targeting of BM-PCa would require tumor-specific delivery to prevent negative effects on the surrounding immune cells.

It is possible that BM-PCa tumors in bone display heterogeneous STAT5 expression, as previously identified in primary PCa tumors with intratumor variability in STAT5 protein levels [33], such that neutrophil targeting would inevitably result in the emergence of a neutrophil-resistant population, similar to acquired drug resistance. There is an additional possibility that neutrophil-resistant PCa may alter long-term neutrophil function. A similar scenario was recently demonstrated in a study of metastatic breast cancer in which IL11-expressing breast cancer cells induced a specific pro-tumoral neutrophil gene signature compared to IL11-null breast tumors which recruited tumor-suppressive neutrophils [34]. As seen with the emergence of acquired drug resistance, future studies are needed to identify specific mechanisms associated with PCa resistance to neutrophil cytotoxicity and the impact of neutrophils on heterogeneous STAT5-expressing BM-PCa tumors.

There is little to no understanding of the contribution of neutrophil/neutrophil precursor populations to tumor growth in the PCa tumor-bone microenvironment. Previously, two different neutrophil subpopulations were identified in lung tumor tissue, namely anti-tumorigenic, N1, and pro-tumorigenic, N2 PMNs, the latter accumulated as a result of increased TGFβ in the tumor microenvironment [12]. These descriptors have been expanded based upon context-specific neutrophil roles [21, 35]. We found that, although BM-PCa increased neutrophil expression of TβRI, this increase had little impact on neutrophil-mediated PCa apoptosis despite increasing neutrophil viability. Depletion of neutrophils in an established bone tumor compared to post-dissemination (recapitulated by depletion 3 days after tumor inoculation) resulted in accelerated tumor growth suggesting that neutrophils in the tumor-bone environment are initially cytotoxic and anti-tumoral. This was further shown by neutrophil activation (i.e., oxidative burst and NET formation) in response to PCa media. However, longitudinal evaluation of neutrophils isolated from the bone marrow throughout tumor progression revealed that bone TANs lose their cytotoxic potential as the tumor progressed. This change in function was associated with reduced neutrophil maturation, evidenced by prolonged viability (characteristic of pre-mitotic neutrophils) and reduced activation/NET formation, characteristics linked to tumor-associated immature neutrophils/granulocytic precursors [36]. Our findings suggest that TANs in the tumor-bone microenvironment initially exhibit characteristics associated with both cytotoxic/N1and granulocytic MDSCs (G-MDSCs) [37] but are functionally altered as the tumor grows via undetermined mechanisms. These findings reveal a need for full characterization of specific neutrophil populations associated with PCa killing within the prostate tumor-bone microenvironment to identify methods to prevent PCa growth by enhancing neutrophil cytotoxicity and/or prevent loss of neutrophil anti-tumoral function.

In these studies, we utilized immunocompromised mice for investigation of human PCa growth in bone. Despite differences that exist between human and mouse neutrophils, we demonstrated that both human and mouse bone marrow-derived neutrophils were cytotoxic to human PCa cells and that our data were not based upon species-specific neutrophil functions. Additionally, we found that bone marrow neutrophils inhibit tumor growth in the bone microenvironment, independently of T cell immunosuppressive functions. Interestingly, cytotoxic TANs isolated from the prostate tumor-bone microenvironment were significantly suppressive of CD4 + T cell proliferation. Although previous studies have shown that neutrophils or granulocytic MDSCs recruited to the primary tumor are associated with increased tumor growth, we present evidence that TANs in the tumor-bone microenvironment can be both tumor-inhibitory and immunosuppressive. In support of our findings, a recent study revealed that mature peripheral blood neutrophils displaying classical cytotoxic effector functions were significantly suppressive of T cell proliferation compared to non-cytotoxic, immature neutrophils [38] demonstrating that cytotoxic neutrophils can suppress T cells. In our studies, both tumor-naïve neutrophils and TANs isolated from the prostate tumor-bone environment were CD11b^+^/Gr-1^hi^, markers associated with G-MDSCs in the tumor microenvironment [39], that displayed cytotoxic effects against PCa. Importantly, therapeutic depletion of these cells may be counterintuitive against treatment of BM-PCa. These findings emphasize the need for characterization of context-specific neutrophil functions and cell surface markers to identify specific effector populations in the tumor microenvironment. Understanding the direct functions of TANs in the bone marrow and their interaction with other immune populations are critical for developing myeloid-targeted therapies to treat bone metastasis.

These studies are the first, to date, to examine direct interactions of BM-PCa and neutrophils, which revealed a role for neutrophils in BM-PCa progression. For prostate cancer patients with metastatic disease, an elevated neutrophil-to-lymphocyte ratio (NLR) has been associated with poor prognosis for survival [40]. Although overall NLR has been shown to be important for disease stratification, it remains unclear whether circulating neutrophils recapitulate neutrophil function in the tumor-bone microenvironment. It is possible that neutrophil mobilization into blood could be induced by PCa in bone and this could be a mechanism of PCa to evade neutrophil-mediated death; however, this possibility has not yet been examined. Our findings demonstrated that bone marrow neutrophils initially will induce death of disseminated PCa cells and lose this impact as the tumor progresses. These results implicate that there may be a specific therapeutic window after PCa dissemination into the bone, where an enhanced neutrophil cytotoxicity could be used to prevent tumor growth. This also suggests that therapies that induce neutropenia, such as chemotherapy, may contribute to disseminated tumor growth in bone. In support of this, a previous study showed that cyclophosphamide enhanced PCa growth in bone via impact on the bone stromal cells, though neutrophils were not investigated [41]. Further, BM-PCa patients typically receive androgen deprivation therapy which could impact immune cell differentiation. Specifically, loss of androgen receptor (AR) inhibits precursor neutrophil proliferation and maturation [42] and it is unclear how this would impact neutrophil function in a clinical setting.

The findings from this study suggest that harnessing neutrophil cytotoxicity in bone by regulating neutrophil maturation and mobilization could prevent PCa growth in bone. For example, granulocyte colony-stimulating factor (G-CSF) is a primary regulator of neutrophil proliferation and differentiation and is often given to cancer patients to replenish the neutrophil population after chemotherapy-related neutropenia [43]. G-CSF mimetics are currently in clinical trial in combination with chemotherapy and could be considered for treatment of BM-PCa [44]. Although we did not examine the role of androgen deprivation therapy on neutrophil function in the prostate tumor-bone microenvironment, ongoing studies are examining the impact of standard of care BM-PCa therapies in the immune–bone microenvironment.

While other preclinical PCa studies highlighted that neutrophils contribute to the progression of prostate tumors, there remains a significant gap in the understanding of neutrophil function in the context of the tumor-bone microenvironment and BM-PCa. Although BM-PCa survival and proliferation in bone is heavily dependent on interactions with and activation of surrounding bone stromal cells, such as osteoclasts and osteoblasts; this is the first study to demonstrate a protective role for neutrophils against BM-PCa via STAT5-mediated PCa death, a phenotype that is altered as the tumor progresses. In summary, neutrophils in bone play a significant role in regulating growth of disseminated PCa and harnessing neutrophil cytotoxic activity in bone could present a novel immunotherapeutic strategy for treating BM-PCa.

## Electronic supplementary material

Below is the link to the electronic supplementary material.

Supplementary material 1 (MP4 3834 kb)

Supplementary material 2 (PDF 93 kb)

Supplementary material 3 (PDF 1102 kb)

## Abbreviations

BM-PCa: Bone metastatic prostate cancer
CM: Conditioned media
DHR 1,2,3: Dihydrorhodamine 1,2,3
HSC: Hematopoietic stem cell
IL-8: Interleukin 8
LPS: Lipopolysaccharide
MDSC: Myeloid-derived suppressor cell
MPO: Myeloperoxidase
MSC: Mesenchymal stem cell
NE: Neutrophil elastase
NET: Neutrophil extracellular trap
PMA: Phorbol 12-myristate 13-acetate
ROS: Reactive oxygen species
TGFβ: Transforming growth factor beta
TβRI: Transforming growth factor beta receptor I
