# Supplementary material for: Neutrophils are mediators of metastatic prostate cancer progression in bone
Source: Cancer Immunol Immunother. 2020 Feb 29;69(6):1113–30. doi: 10.1007/s00262-020-02527-6 (PMC7230043; doi:10.1007/s00262-020-02527-6)
Supplement: Supplementary file 2 — Supplementary material 2 (PDF 93 kb) [file 262_2020_2527_MOESM2_ESM.pdf]

Supplemental Table 1. List of RealTime qPCR primers.

Supplemental Figure 1. Neutrophil localization in BM-PCa patient specimens. Representative IF image of (a) neutrophil elastase-red and pan-cytokeratin-green in Patients 1,3 and 4 or neutrophil elastase-green, myeloperoxidase-red, pan-cytokeratin-gold in Patients 5-7, and nuclear marker DAPI-blue (all images). (b) Representative image of cytokeratin-positive (green) C42B cells in mouse tibia. Bone marrow neutrophils are identified by neutrophil elastase (red). Size bar = 50μm.

Supplemental Figure 2. BM-PCa induces neutrophil oxidative burst. Scatter plots of DHR oxidation (green fluorescence) measured by flow cytometry of viable neutrophils treated with: conditioned serum-free media from benign prostatic hyperplasia (BPH-1), LNCaP, C42B, PC3. For comparison, neutrophils were treated with complete RPMI (negative control) or RPMI supplemented with 5nM PMA (positive control) (b) A separate experiment of longitudinal measurement of DHR oxidation/green fluorescence by Incucyte S3 Live Cell imager.

Supplemental Figure 3. Confluency rates of neutrophil-PCa co-cultures. Growth rate analysis of direct co-culture with mouse neutrophils using the Incucyte Live Cell imager in LNCaP and C42B cells (n=3-4 for each condition). Neutrophils were added 24 hours after plating PCa cells and the plate was added to the Incucyte for imaging. Percent confluence was calculated by normalizing cell numbers at each time point to time 0/the start of the experiment.

Supplemental Figure 4. TGFβ regulates neutrophil viability but has little impact on neutrophil-mediated PCa death. Mouse and human neutrophils were isolated from bone marrow and treated for 3 hours with LNCaP and C42B CM (n= 3). (a), (d) Graph shows fold change of *TGFβ*, *TβRI* and *TβRII* expression in C42B-treated neutrophils compared to LNCaP-treated neutrophils. 18S was used as an endogenous control. Asterisks denote statistical significance (*$p<0.05$, ***$p<0.001$). (b),(e) Human neutrophils were isolated from bone marrow and reconstituted in LNCaP or C42B CM in supplemented with an ALK5 inhibitor (RepSox, denoted as ALK5i;5nM). Luminescence was measured at specific time points as a readout for neutrophil viability using RealTime Glo MT viability assay. Graph represents the change in luminescence normalized to Day 0 (100%) when neutrophils were reconstituted in appropriate conditions. Asterisks denote statistical significance (*$p<0.05$, ****$p<0.0001$) at 48 hours. (c) Direct co-culture of LNCaP (left) and C42B (right) cells in the presence or absence of an ALK5 inhibitor (RepSox, 5nM). (n=3 for each) Asterisks denote statistical significance (*$p<0.05$)

Supplemental Figure 5. Anti-Ly6G efficiently depletes neutrophil populations in the tibia. Flow cytometry was performed on whole bone marrow flushes (a) and spleen (b) from tumor-bearing tibia of isotype control and anti-Ly6G antibody. Live cells were gated by CD45+/ CD11b+ (for myeloid leukocytes) and the percent of Ly6C and Ly6G expressing cells examined in bone. For spleen, Ly6G and Gr-1 was measured.

Supplemental Figure 6. Bone marrow TANs are altered throughout tumor progression. C42B (or saline as a control) were injected into tibia of male SCID Beige mice (n=4) and neutrophils were isolated from bone at Weeks 1, 2, and 4 after injection. (a) Luminescence (using viability dye RealTime Glo) of TANs compared to control neutrophils. Asterisks denote statistical significance (****p<0.0001*) (b) NET formation is shown using percentage of Sytox Green-positive NET DNA per total cell number. (c) T cell proliferation assay of naïve CD4+ T cells incubated with control neutrophils and TANs isolated from tumor-bone marrow.

Supplemental Figure 7. Neutrophil impact on PCa receptor tyrosine kinase phosphorylation. (a) Quantification of phosphorylation status of 46 proteins on the Human Phospho-Kinase protein array. Phospho-Kinase array was performed using lysates from a direct co-culture of neutrophils with cancer cells. Densitometry analysis was performed in Image J software. Background wells were subtracted from dot values, and normalized to the reference control dots. Asterisks denote statistical significance (**p<0.05*, ***p<0.01, ***p<0.001, ****p<0.0001*). (b) C42B (left) and LNCaP (right) cell counts 24 hours after direct or indirect co-culture with mouse neutrophils. (c) Western blot analysis of Stat5 in PC3M compared to PAIII. (d) PC3M cell counts 24 hours after direct co-culture with human bone marrow neutrophils.

Supplemental Figure 8. STAT5A knockdown in C42B PCa cells. (a) RealTime qPCR analysis of *STAT5A* and *STAT5B* in C42B cells 24 hours after culture with mouse neutrophils. Graph shows expression relative to *18S* and normalized to C42B only (no neutrophils). Asterisk denotes statistical significance (**p<0.05*). (b) C42B were transfected with a red fluorescent protein (RFP)-expressing STAT5 shRNA (or scrambled control sequence) and purified via FACS. Representative phase contrast and RFP images (left) of C42B STAT5A control and knockdown (KD) cells. Graph (right) shows quantitation of fluorescence in STAT5 KD C42B clones compared to parental luciferase-expressing C42B. (c) RealTime qPCR of STAT5A and STAT5B gene expression in STAT5A KD C42B relative to Scrambled Control C42B. 18S was used as an endogenous control. Asterisks denote statistical significance (**p<0.05,**p<0.01,****p<0.00001* (d) Western blot of STAT5 and STAT3 protein

expression in C42B Scrambled control and STAT5 knockdown C42B. Western Blot image (left) and densitometry (right) of STAT proteins (STAT5-black bar; STAT3-red bar) normalized to total beta actin.

Supplemental Movie 1. Neutrophil contact with C42B cells. Mouse bone marrow neutrophils were isolated and directly added to C42B cultures. C42B cells were plated 24 hours prior to the addition of Live video was taken over one hour using 3-D NanoLive holotomographic microscopy, as described[20]. Video shows three neutrophils directly contacting the C42B cell.
