# Supplementary material for: Neutrophils are mediators of metastatic prostate cancer progression in bone
Source: Cancer Immunol Immunother. 2020 Feb 29;69(6):1113–30. doi: 10.1007/s00262-020-02527-6 (PMC7230043; doi:10.1007/s00262-020-02527-6)
Supplement: Supplementary file 3 — Supplementary material 3 (PDF 1102 kb) [file 262_2020_2527_MOESM3_ESM.pdf]

Supplemental Table 1.RealTime qPCR primers

Mouse primers	sequence
TGFBR1	mTβR1-FWD 5'-CGCTCTGTCCACGGCAAG-3'<br>mTβR1-RV 5'-TCATGTCTCACAGCAAGTCCC-3'
TGFBR2	mTβR2-FWD 5'-GGCCAAGCTGAAGCAGAA-3'<br>mTβR2-RV 5'-GGATGTTCTCGTGTTTCAGGTT-3'
TGFB1	mTGFβ1-FWD 5'-CTCCCGTGGCTTCTAGTG-3'<br>mTGFβ1 RV 5'-GCCTTAGTTTGGACAGGATCTG-3'

Human primers	sequence
TGFBR2	huTβR2 FWD 5'-ACTTTATTCTGGAAGCTGCT-3'<br>huTβR2 RV 5'-GCTGATGCCTGTCACTTGAA-3'
TGFBR1	huTGFβ1 FWD 5'-CCCAGCATCTGCAAAGCTC-3'<br>huTGFβ1 RV 5'-GTCAATGTACAGCTGCCGCA-3'
TGFB1	huTβR1 FWD 5'-CGAGTGCCAAATGAAGAGGA-3'<br>huTβR1 RV 5'-CGACCTTTGCCAATGCTTTC-3'
STAT5A	huSTAT5A FWD 5'-TTACTGAAGATCAAGCTGGGG-3'<br>huSTAT5A RV 5'-ATTGTACAGAATGTGCCGG-3'
STAT5B	huSTAT5B FWD 5'-CATTTTCCCATTGAGGTGC-3'<br>huSTAT5B RV 5'-GGGTGGCCTTAATGTTCTCC-3'
IL8	hu IL8 FWD 5'-CTTGGCAGCCTTCCTGATTT-3'<br>hu IL8 REV 5'-TTCTTTAGCACTCCTTGGCAA-3'
18s	18s FWD 5'-GTAACCCGTTGAACCCCATT-3'<br>18S RV 5'-CCATCCAATCGGTAGTAGCG-3'

Supplemental Figures

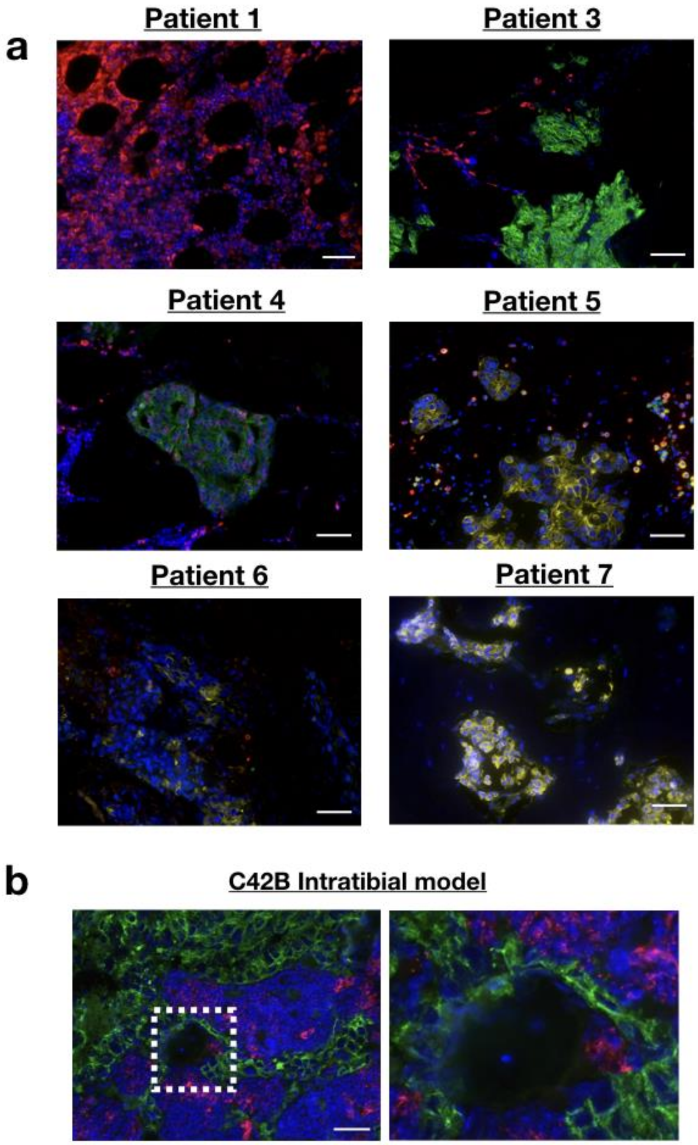

Costanzo-Garvey et al. Supp. Figure 1

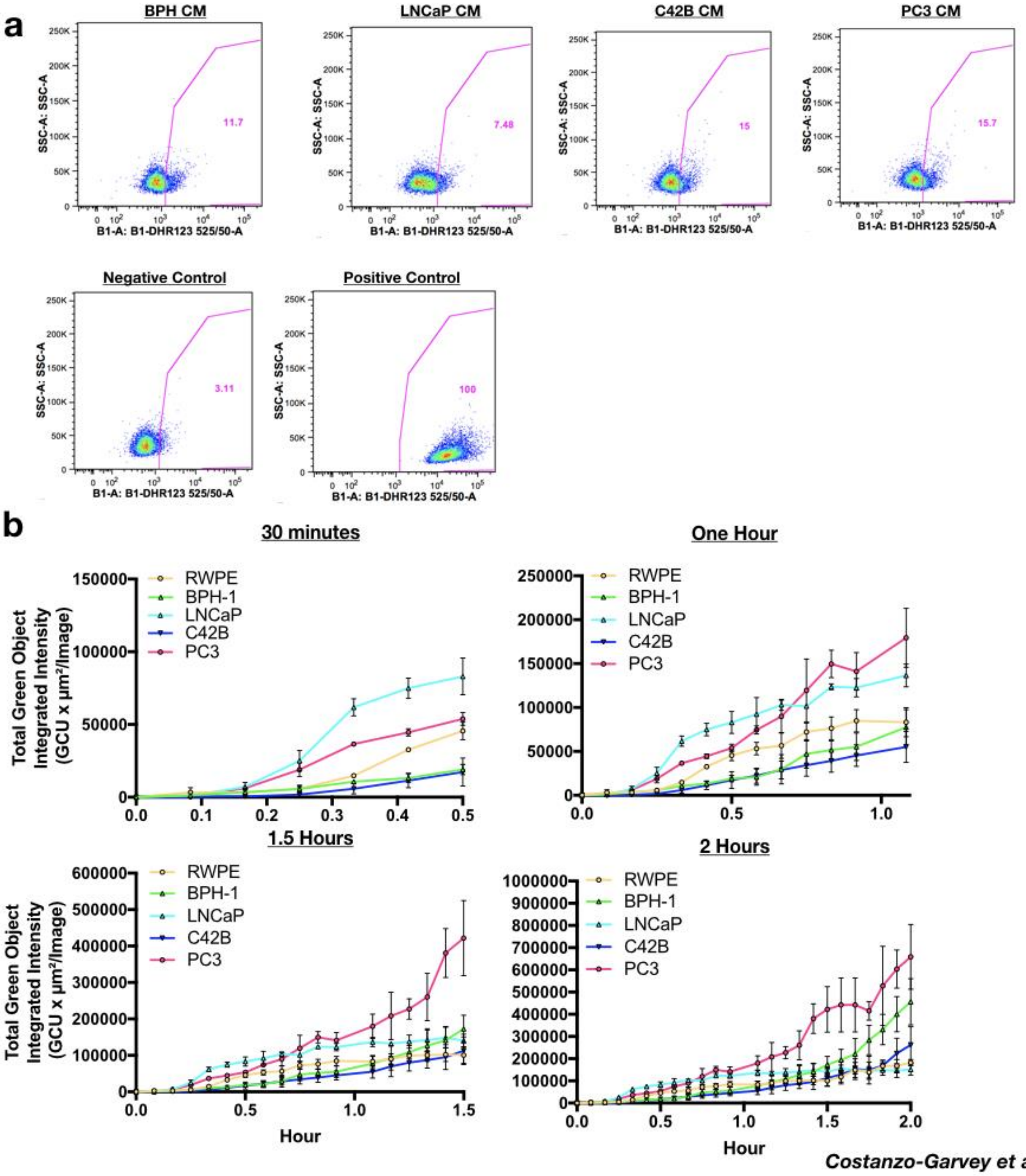

Costanzo-Garvey et al. Supp Figure 2

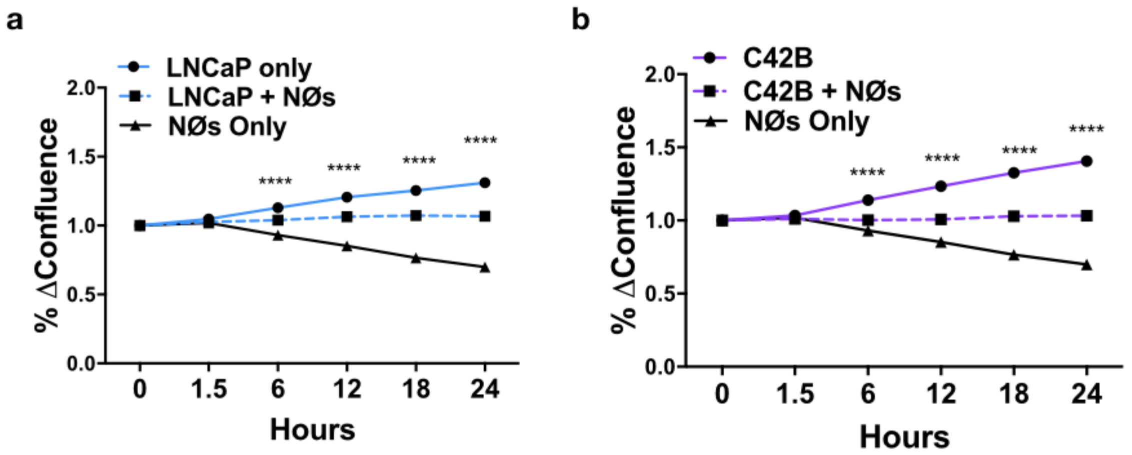

Costanzo-Garvey et al. Supp. Figure 3

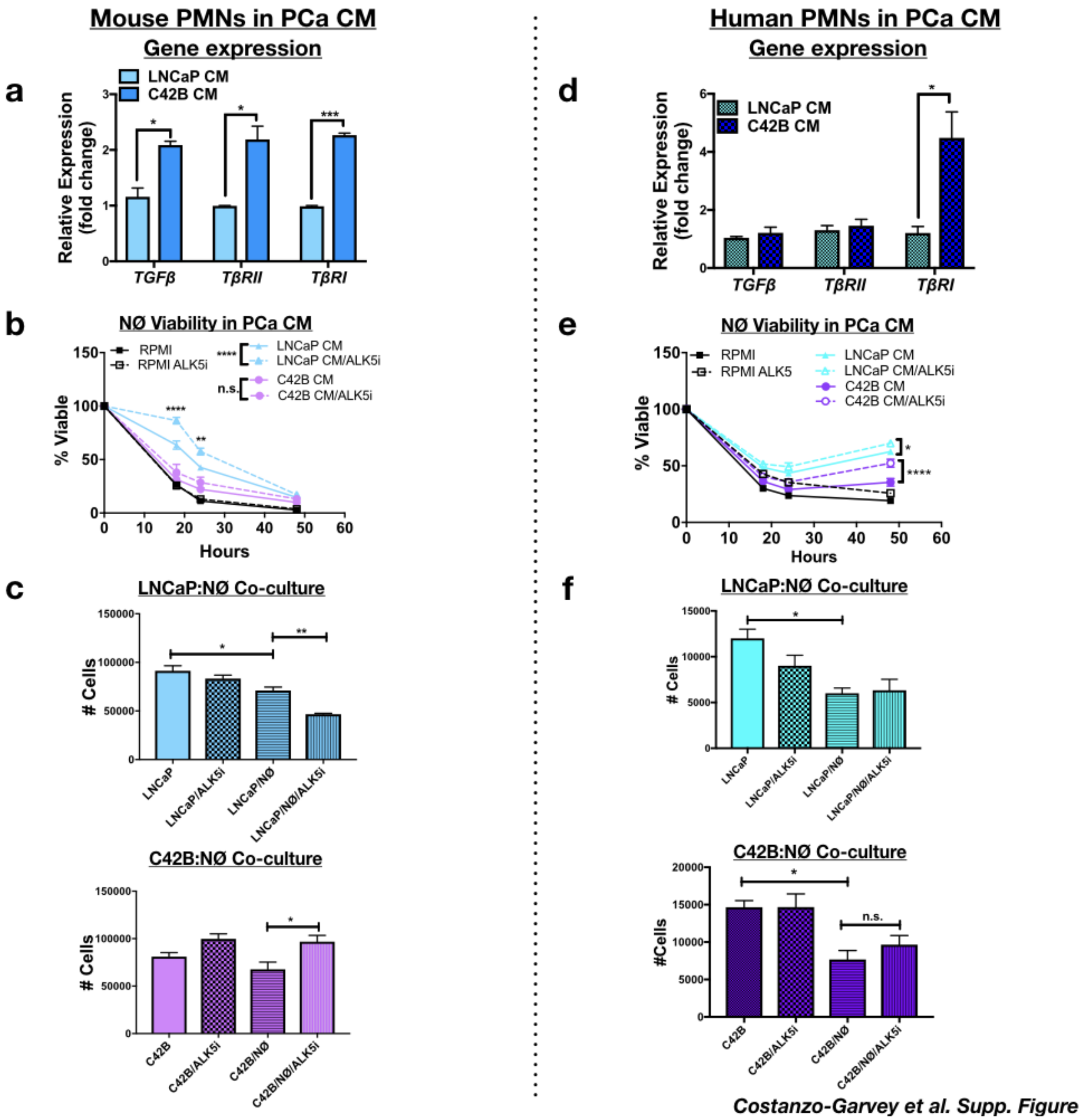

Costanzo-Garvey et al. Supp. Figure 4

a **Bone Marrow**

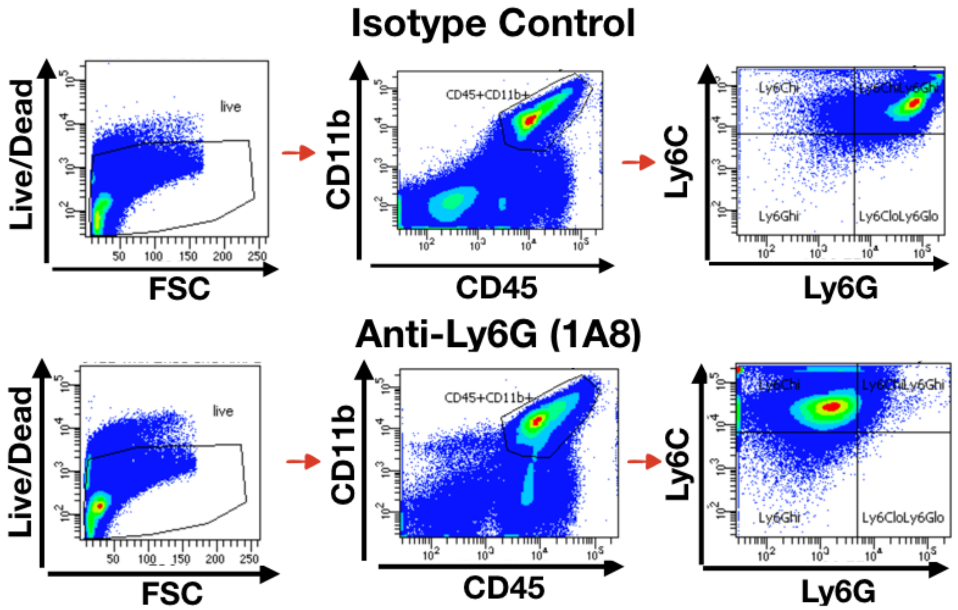

b **Spleen**

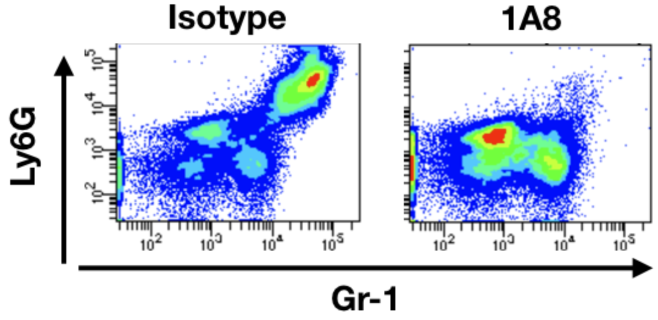

Costanzo-Garvey et al. Supp. Figure 5

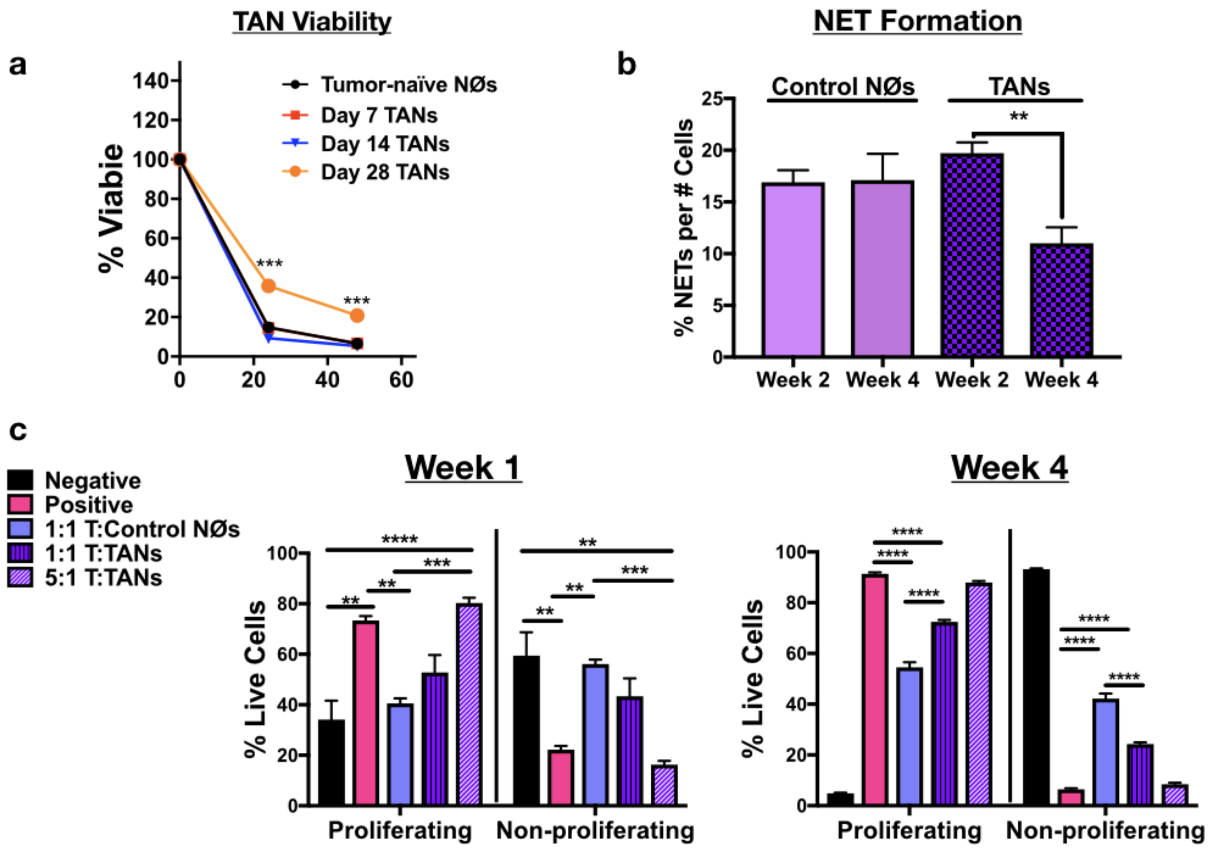

Costanzo-Garvey et al. Supp. Figure 6

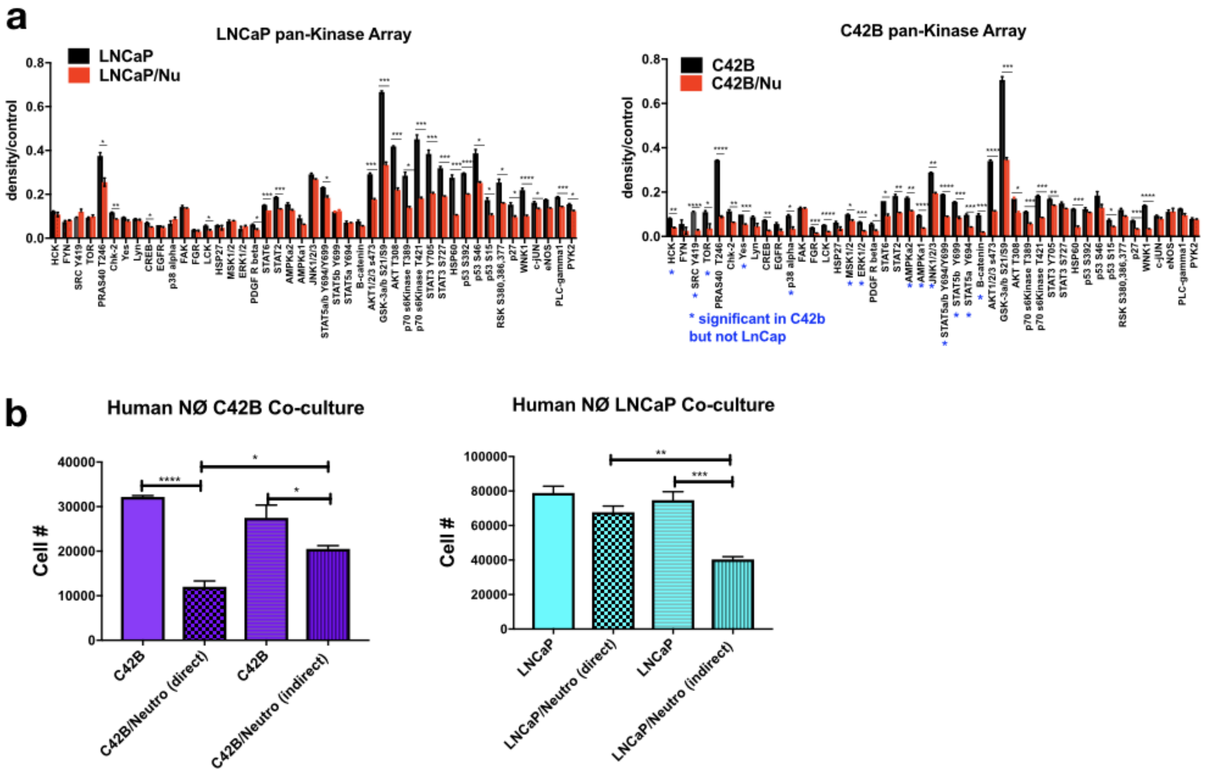

Costanzo-Garvey et al. Supp. Figure 7

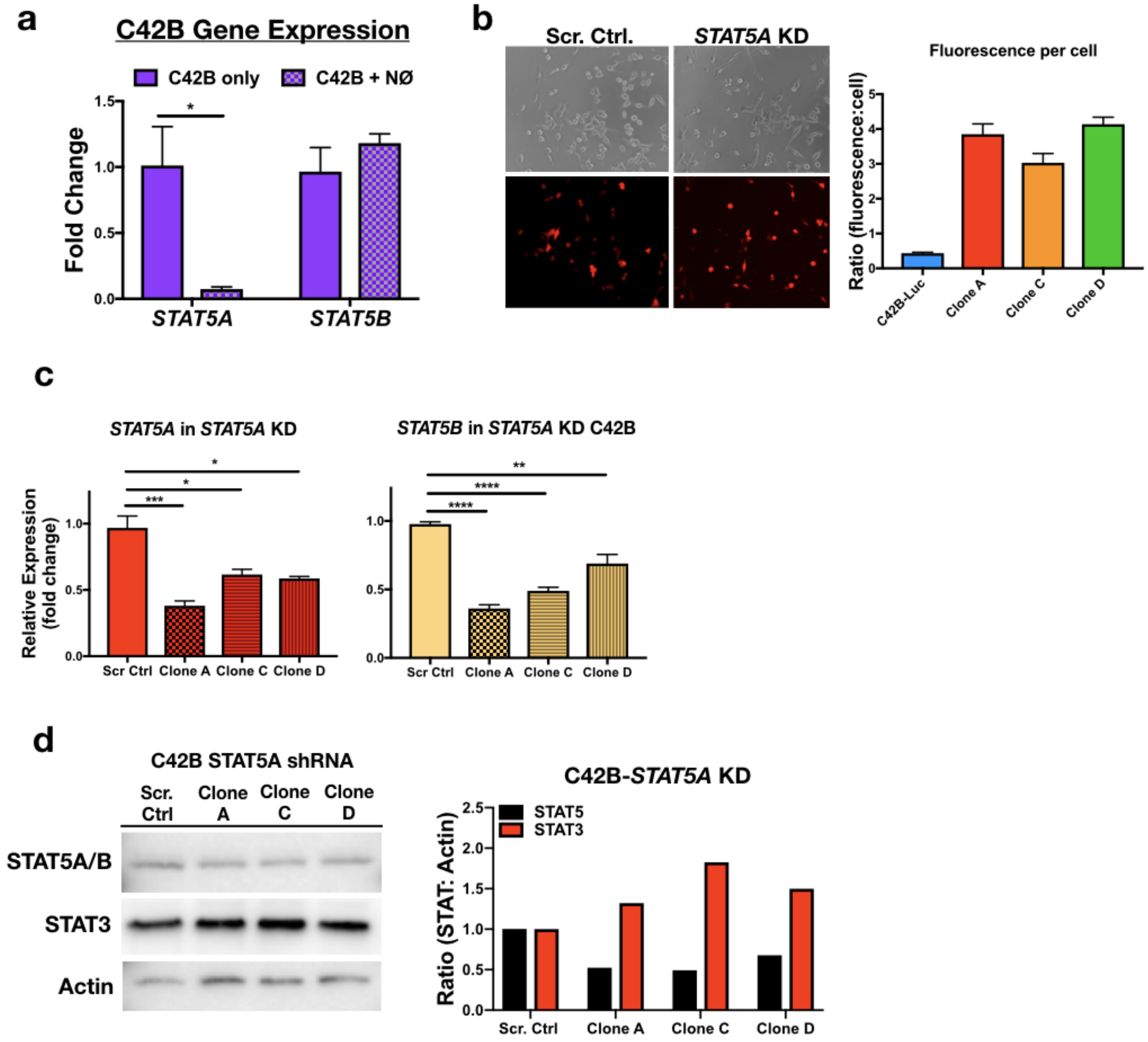

Costanzo-Garvey et al. Supp Figure 8
